# On Event-Triggered Adaptive Architectures for Decentralized and Distributed Control of Large-Scale Modular Systems

**DOI:** 10.3390/s16081297

**Published:** 2016-08-16

**Authors:** Ali Albattat, Benjamin C. Gruenwald, Tansel Yucelen

**Affiliations:** 1 Department of Mechanical and Aerospace Engineering, Missouri University of Science and Technology, Rolla, MO 65409, USA; ata365@mst.edu; 2 Laboratory for Autonomy, Control, Information, and Systems (LACIS), Department of Mechanical Engineering, University of South Florida, Tampa, FL 33620, USA; bcgruenwald@mail.usf.edu

**Keywords:** large-scale modular systems, networked control systems, uncertain dynamical systems, event-triggered control, decentralized control, distributed control, system stability and performance

## Abstract

The last decade has witnessed an increased interest in physical systems controlled over wireless networks (networked control systems). These systems allow the computation of control signals via processors that are not attached to the physical systems, and the feedback loops are closed over wireless networks. The contribution of this paper is to design and analyze event-triggered decentralized and distributed adaptive control architectures for uncertain networked large-scale modular systems; that is, systems consist of physically-interconnected modules controlled over wireless networks. Specifically, the proposed adaptive architectures guarantee overall system stability while reducing wireless network utilization and achieving a given system performance in the presence of system uncertainties that can result from modeling and degraded modes of operation of the modules and their interconnections between each other. In addition to the theoretical findings including rigorous system stability and the boundedness analysis of the closed-loop dynamical system, as well as the characterization of the effect of user-defined event-triggering thresholds and the design parameters of the proposed adaptive architectures on the overall system performance, an illustrative numerical example is further provided to demonstrate the efficacy of the proposed decentralized and distributed control approaches.

## 1. Introduction

The design and implementation of decentralized and distributed architectures for controlling complex, large-scale systems is a nontrivial control engineering task involving the consideration of components interacting with the physical processes to be controlled. In particular, large-scale systems are characterized by a large number of highly coupled components exchanging matter, energy or information and have become ubiquitous given the recent advances in embedded sensor and computation technologies. Examples of such systems include, but are not limited to, multi-vehicle systems, communication systems, power systems, process control systems and water systems (see, for example, [1,2,3,4,5,6] and the references therein). This paper concentrates on an important class of large-scale systems; namely, large-scale modular systems that consist of physically-interconnected and generally heterogeneous modules.

### 1.1. Motivation and Literature Review

Two sweeping generalizations can be made about large-scale modular systems. The first is that their complex structure and large-scale nature yield to inaccurate mathematical module models, since it is a challenge to precisely model each module of a large-scale system and the interconnections between these modules. As a consequence, the discrepancies between the modules and their mathematical models, that is system uncertainties, result in the degradation of overall system stability and the performance of the large-scale modular systems. To this end, adaptive control methodologies [7,8,9,10,11,12,13] offer an important capability for this class of dynamical systems to learn and suppress the effect of system uncertainties resulting from modeling and degraded modes of operation, and hence, they offer system stability and desirable closed-loop system performance in the presence of system uncertainties without excessively relying on mathematical models.

The second generalization about large-scale modular systems is that these systems are often controlled over wireless networks, and hence, the communication costs between the modules and their remote processors increase proportionally with the increase in the number of modules and often the interconnection between these modules. To this end, event-triggered control methodologies [14,15,16] offer new control execution paradigms that relax the fixed periodic demand of computational resources and allow for the aperiodic exchange of sensor and actuator information with the remote processor to reduce overall communication cost over a wireless network. Note that adaptive control methodologies and event-triggered control methodologies are often studied separately in the literature, where it is of practical importance to theoretically integrate these two approaches to guarantee system stability and the desirable closed-loop system performance of uncertain large-scale modular systems with reduced communication costs over wireless networks, which is the main focus of this paper.

More specifically, the authors of [6,17,18,19,20,21,22,23] proposed decentralized and distributed adaptive control architectures for large-scale systems; however, these approaches do not make any attempts to reduce the overall communication cost over wireless networks using, for example, event-triggered control methodologies. In addition, the authors of [24,25,26,27,28,29,30] present decentralized and distributed control architectures with event triggering; however, these approaches do not consider adaptive control architectures and assume perfect models of the processes to be controlled; hence, they are not practical for large-scale modular systems with significant system uncertainties. Only the authors of [31,32,33,34,35,36] present event-triggered adaptive control approaches for uncertain dynamical systems. In particular, the authors of [31,32] consider data transmission from a physical system to the controller, but not vice versa, while developing their adaptive control approaches to deal with system uncertainties. On the other hand, the adaptive control architectures of the authors in [33,34,35,36] consider two-way data transmission over wireless networks; that is, from a physical system to the controller and from the controller to this physical system. However, none of these approaches can be directly applied to large-scale modular systems. This is due to the fact that large-scale modular systems require decentralized and distributed architectures, and direct application of the results in [31,32,33,34,35,36] to this class of systems can result in centralized architectures, which is not practically desired due to the large-scale nature of modular systems. To summarize, there do not exist resilient adaptive control architectures for large-scale systems in the literature to deal with system uncertainties while reducing the communication costs between the models and their remote processors.

### 1.2. Contribution

The contribution of this paper is to design and analyze event-triggered decentralized and distributed adaptive control architectures for uncertain large-scale systems controlled over wireless networks. Specifically, the proposed decentralized and distributed adaptive architectures of this paper guarantee overall system stability while reducing wireless network utilization and achieving a given system performance in the presence of system uncertainties that can result from modeling and degraded modes of operation of the modules and their interconnections between each other. From a theoretical viewpoint, the proposed event-triggered adaptive architectures here can be viewed as a significant generalization of our prior work documented in [35,36] to large-scale modular systems, which consider a state emulator-based adaptive control methodology with robustness against high-frequency oscillations in the controller response [10,13,37,38,39,40,41,42]. In this generalization, we also adopt necessary tools and methods from [6,23] on decentralized and distributed adaptive controller construction for large-scale modular systems. In addition to the theoretical findings including rigorous system stability and boundedness analysis of the closed-loop dynamical system and the characterization of the effect of user-defined event-triggering thresholds, as well as the design parameters of the proposed adaptive architectures on the overall system performance, an illustrative numerical example is further provided to demonstrate the efficacy of the proposed decentralized and distributed control approaches.

### 1.3. Organization

The contents of the paper are as follows. In Section 2, we consider an event-triggered decentralized adaptive control approach for large-scale modular systems, where the considered approach assumes that physically-interconnected modules cannot communicate with each other for exchanging their state information. Specifically, Theorem 1 and Corollaries 1–4 show the main results of Section 2 subject to some structural conditions on the parameters of the large-scale modular systems and the proposed event-triggered decentralized control architecture (see Assumptions 4 and 5). In Section 3, we consider an event-triggered distributed adaptive control approach in Theorem 2 and Corollaries 5–7 for getting rid of such structural conditions, where the considered approach assumes that physically-interconnected modules can locally communicate with each other for exchanging their state information. Finally, the illustrative numerical example is presented in Section 4, and conclusions are summarized in Section 5.

### 1.4. Notation

The notation used in this paper is fairly standard. Specifically, R denotes the set of real numbers; $R^{n}$ denotes the set of n×1 real column vectors; $R^{n\times m}$ denotes the set of n×m real matrices; $R_{+}$ denotes the set of positive real numbers; $R_{+}^{n\times n}$ denotes the set of n×n positive-definite real matrices; $S^{n\times n}$ denotes the set of n×n symmetric real matrices; $D^{n\times n}$ denotes the set of n×n real matrices with diagonal scalar entries; ${\left(\cdot \right)}^{T}$ denotes transpose; ${\left(\cdot \right)}^{-1}$ denotes inverse; tr(·) denotes the trace operator; diag(a) denotes the diagonal matrix with the vector *a* on its diagonal; and “≜” denotes equality by definition. In addition, we write $\lambda_{\text{min}}\left(A\right)$ (respectively, $\lambda_{\text{max}}\left(A\right))$ for the minimum and respectively maximum eigenvalue of the Hermitian matrix *A*, ∥·∥ for the Euclidean norm and ${∥\cdot ∥}_{F}$ for the Frobenius matrix norm. Furthermore, we use “∨” for the “or” logic operator and “$\bar{\left(\cdot \right)}$” for the “not” logic operator.

We adopt graphs [43] to encode physical interactions and communications between modules. In particular, an undirected graph G is defined by $V_{G}=\left\{1,\cdots ,N\right\}$ of nodes and a set $E_{G}\in V_{G}\times V_{G}$ of edges. If $\left(i,j\right)\in E_{G},$ then the nodes *i* and *j* are neighbors, and the neighboring relation is indicated with i∼j. The degree of a node is given by the number of its neighbors, where $d_{i}$ denotes the degree of node *i*. Lastly, the adjacency matrix of a graph G, $A\left(G\right)\in R^{N\times N}$, is given by:

(1) $${\left[A(G)\right]}_{ij}≜\left\{\begin{array}{cc} & 1,\;\;\text{if}\;\left(i,j\right)\in E_{G}\\ & 0,\;\text{otherwise} \end{array}\right.$$

## 2. Event-Triggered Decentralized Adaptive Control

In this section, we introduce an event-triggered decentralized adaptive control architecture, where it is assumed that physically-interconnected modules cannot communicate with each other. For organizational purposes, this section is broken up into two subsections. Specifically, we first briefly overview a standard decentralized adaptive control architecture without event-triggering and then present the proposed event-triggered decentralized adaptive control approach, which includes rigorous stability and performance analyses with no Zeno behavior and generalizations to the state emulator case for suppressing the effect of possible high-frequency oscillations in the controller response.

### 2.1. Overview of a Standard Decentralized Adaptive Control Architecture without Event-Triggering

Consider an uncertain large-scale modular system S consisting of *N* interconnected modules $S_{i}$, $i\in V_{G}$, given by:

(2) $$S_{i}:\;{\dot{x}}_{i}\left(t\right)=A_{i}x_{i}\left(t\right)+B_{i}\left[\Lambda_{i}u_{i}\left(t\right)+\Delta_{i}\left(x_{i}\left(t\right)\right)+\sum_{i∼j}\delta_{ij}\left(x_{j}\left(t\right)\right)\right],\;x_{i}\left(0\right)=x_{i0}$$

where $x_{i}\left(t\right)\in R^{n_{i}}$ is the state of $S_{i}$, $u_{i}\left(t\right)\in R^{m_{i}}$ is the control input applied to $S_{i}$, $A_{i}\in R^{n_{i}\times n_{i}}$, $B_{i}\left(t\right)\;\in \;R^{n_{i}\times m_{i}}$ are known matrices and the pair $\left(A_{i},B_{i}\right)$ is controllable. In addition, $\Lambda_{i}\in R_{+}^{m_{i}\times m_{i}}\cap D^{m_{i}\times m_{i}}$ is an unknown module control effectiveness matrix; $\Delta_{i}:R^{n_{i}}\to R^{m_{i}}$ represents matched module bounded uncertainties; and $\delta_{ij}:R^{n_{j}}\to R^{m_{i}}$ represents matched unknown physical interconnections with respect to module *j*, $j\in V_{G}$, such that $\left(i,j\right)\in E_{G}$.

**Assumption 1.**  *The unknown module uncertainty is parameterized as*:

(3) $$\begin{array}{c} \Delta_{i}\left(x_{i}\left(t\right)\right)=W_{oi}^{T}\beta_{i}\left(x_{i}\left(t\right)\right),\;x_{i}\in R^{n_{i}} \end{array}$$

where $W_{oi}\in R^{g_{i}\times m_{i}}$ is an unknown weight matrix, which satisfies $∥W_{oi}{∥}_{F}\leq \omega_{i}^{*}$, $\omega_{i}^{*}\in R_{+}$, and $\beta_{i}\left(x_{i}\left(t\right)\right):R^{n_{i}}\to R^{g_{i}}$ is a known Lipschitz continuous basis function vector satisfying:

(4) $$\begin{array}{c} ∥\beta_{i}\left(x_{1i}\right)-\beta_{i}\left(x_{2i}\right)∥\leq L_{\beta i}∥x_{1i}-x_{2i}∥ \end{array}$$

*with $L_{\beta i}\in R_{+}$.*

**Assumption 2.**  *The function $\delta_{ij}\left(x_{j}\left(t\right)\right)$ in Equation (2) satisfies:*

(5) $$\begin{array}{c} ∥\delta_{ij}\left(x_{j}\left(t\right)\right)∥\leq \alpha_{ij}∥x_{j}\left(t\right)∥,\;\alpha_{ij}>0,\;x_{j}\in R^{n_{j}} \end{array}$$

Next, consider the reference model $S_{ri}$ capturing a desired closed-loop performance for module *i*, $i\in V_{G}$ given by:

(6) $$\begin{array}{c} S_{ri}:\;{\dot{x}}_{ri}\left(t\right)=A_{ri}x_{ri}\left(t\right)+B_{ri}c_{i}\left(t\right),\;x_{ri}\left(0\right)=x_{ri0} \end{array}$$

where $x_{ri}\left(t\right)\in R^{n_{i}}$ is the reference state vector of $S_{ri}$, $c_{i}\left(t\right)\in R^{m_{i}}$ is a given bounded command of $S_{ri}$, $A_{ri}\in R^{n_{i}\times n_{i}}$ is the reference system matrix and $B_{ri}\in R^{n_{i}\times m_{i}}$ is the command input matrix.

**Assumption 3.**  There exist $K_{1i}\in R^{m_{i}\times n_{i}}$ and $K_{2i}\in R^{m_{i}\times m_{i}}$, such that $A_{ri}=A_{i}-B_{i}K_{1i}$ and $B_{ri}=B_{i}K_{2i}$ hold with $A_{ri}$ being Hurwitz.

Using Assumptions 1 and 3, Equation (2) can be equivalently written as:

(7) $$\begin{array}{c} {\dot{x}}_{i}\left(t\right)=A_{ri}x_{i}\left(t\right)+B_{ri}c_{i}\left(t\right)+B_{i}\Lambda_{i}\left[u_{i}\left(t\right)+W_{i}^{T}\sigma_{i}\left(x_{i}\left(t\right),c_{i}\left(t\right)\right)\right]+B_{i}\sum_{i∼j}\delta_{ij}\left(x_{j}\left(t\right)\right) \end{array}$$

where $W_{i}≜{\left[\Lambda_{i}^{-1}W_{oi}^{T}\;,\;\Lambda_{i}^{-1}K_{1i}^{T}\;,\;\Lambda_{i}^{-1}K_{2i}^{T}\right]}^{T}\in R^{\left(g_{i}+n_{i}+m_{i}\right)\times m_{i}}$ is the unknown weight matrix and $\sigma_{i}\left(x_{i}\left(t\right),c_{i}\left(t\right)\right)≜{\left[\beta_{i}^{T}\left(x_{i}\left(t\right)\right)\;,\;x_{i}^{T}\left(t\right)\;,\;c_{i}^{T}\left(t\right)\right]}^{T}\in R^{g_{i}+n_{i}+m_{i}}$. Motivated from the structure of the uncertain terms appearing in Equation (7), let the decentralized adaptive feedback controller of $S_{i},\;i\in V_{G}$, be given by:

(8) $$\begin{array}{c} C_{i}:\;u_{i}\left(t\right)≜-{\hat{W}}_{i}{\left(t\right)}^{T}\sigma_{i}\left(x_{i}\left(t\right),c_{i}\left(t\right)\right) \end{array}$$

where ${\hat{W}}_{i}\left(t\right)$ is an estimate of $W_{i}$ satisfying the update law:

(9) $$\begin{array}{c} {\dot{\hat{W}}}_{i}\left(t\right)≜\gamma_{i}{\mathrm{Proj}}_{m}[{\hat{W}}_{i}\left(t\right)\;,\;\sigma_{i}\left(x_{i}\left(t\right),c_{i}\left(t\right)\right){\left(x_{i}\left(t\right)-x_{ri}\left(t\right)\right)}^{T}P_{i}B_{i}],\;{\hat{W}}_{i}\left(0\right)={\hat{W}}_{i0} \end{array}$$

where ${\mathrm{Proj}}_{m}$ denotes the projection operator defined for matrices [10,35,44,45], $\gamma_{i}\in R_{+}$ being the learning rate and $P_{i}\in R_{+}^{n_{i}\times n_{i}}\cap S^{n_{i}\times n_{i}}$ being a solution of the Lyapunov equation:

(10) $$\begin{array}{c} 0=A_{ri}^{T}P_{i}+P_{i}A_{ri}+R_{i} \end{array}$$

with $R_{i}\in R_{+}^{n_{i}\times n_{i}}\cap S^{n_{i}\times n_{i}}$. Now, letting:

(11) $$\begin{array}{cc} e_{i}\left(t\right) & ≜x_{i}\left(t\right)-x_{ri}\left(t\right) \end{array}$$

(12) $$\begin{array}{cc} \;{\tilde{W}}_{i}\left(t\right) & ≜{\hat{W}}_{i}\left(t\right)-W_{i} \end{array}$$

and using Equations (6) and (7), the module-level closed-loop error dynamics are given by:

(13) $$\begin{array}{c} {\dot{e}}_{i}\left(t\right)=A_{ri}e_{i}\left(t\right)-B_{i}\Lambda_{i}{\tilde{W}}_{i}^{T}\left(t\right)\sigma_{i}\left(x_{i}\left(t\right),c_{i}\left(t\right)\right)+B_{i}\sum_{i∼j}\delta_{ij}\left(x_{j}\left(t\right)\right),\;e_{i}\left(t\right)=e_{i0} \end{array}$$

### 2.2. Proposed Event-Triggered Decentralized Adaptive Control Architecture

**Figure 1:**
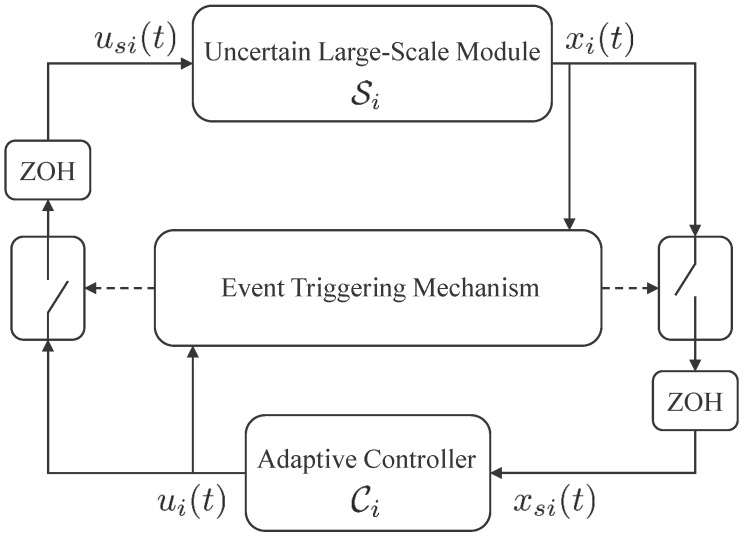
Event-triggered adaptive control for large-scale modular systems.

We now present the proposed event-triggered decentralized adaptive control architecture for large-scale modular systems, which reduces wireless network utilization and allows a desirable command tracking performance during the two-way data exchange between the module $S_{i}$, $i\in V_{G}$, and its local controller $C_{i}$, over a wireless network. For this objective, we utilize event-triggering control theory to schedule the data exchange dependent on errors exceeding user-defined thresholds. Specifically, the module sends its state signal to its local adaptive controller only when a predefined event occurs. The $k_{i}$-th time instants of the state transmission of the module are represented by the monotonic sequence ${\left\{s_{k_{i}}\right\}}_{k_{i}=1}^{\infty }$, where $s_{k_{i}}\in R_{+}$. The local controller uses this triggered module state signal to compute the control signal using adaptive control architecture. In addition, the local controller sends the updated feedback control input to the module only when another predefined event occurs. The $j_{i}$-th time instants of the feedback control transmission are then represented by the monotonic sequence ${\left\{r_{j_{i}}\right\}}_{j_{i}=1}^{\infty }$, where $r_{j_{i}}\in R_{+}$. As depicted in Figure 1, each module state signal and its local control input are held by a zero-order-hold operator (ZOH) until the next triggering event for the corresponding signal takes place. The delay in sampling, data transmission and computation is not considered in this paper. Consider the uncertain dynamical module *i* given by:

(14) $$\begin{array}{c} S_{i}:\;{\dot{x}}_{i}\left(t\right)=A_{i}x_{i}\left(t\right)+B_{i}\left[\Lambda_{i}u_{si}\left(t\right)+\Delta_{i}\left(x_{i}\left(t\right)\right)+\sum_{i∼j}\delta_{ij}\left(x_{j}\left(t\right)\right)\right],\;x_{i}\left(0\right)=x_{i0} \end{array}$$

where $u_{si}\left(t\right)\in R^{m_{i}}$ is the sampled control input vector. Using Assumptions 1 and 3, Equation (14) can be equivalently written as:

(15) $$\begin{array}{ccc} {\dot{x}}_{i}\left(t\right) & = & A_{ri}x_{i}\left(t\right)+B_{ri}c_{i}\left(t\right)+B_{i}\Lambda_{i}\left[u_{si}\left(t\right)+W_{i}^{T}\sigma_{i}\left(x_{i}\left(t\right),x_{si}\left(t\right),c_{i}\left(t\right)\right)\right]+B_{i}\sum_{i∼j}\delta_{ij}\left(x_{j}\left(t\right)\right)\\ & & +B_{i}\Lambda_{i}\left(u_{si}\left(t\right)-u_{i}\left(t\right)\right)+B_{i}K_{1i}\left(x_{si}\left(t\right)-x_{i}\left(t\right)\right) \end{array}$$

where $x_{si}\left(t\right)\in R^{n_{i}}$ is the sampled state vector, $\sigma_{i}\left(x_{i}\left(t\right),x_{si}\left(t\right),c_{i}\left(t\right)\right)≜{\left[\beta_{i}^{T}\left(x_{i}\left(t\right)\right)\;,\;x_{si}^{T}\left(t\right)\;,\;c_{i}^{T}\left(t\right)\right]}^{T}\in R^{g_{i}+n_{i}+m_{i}}$. Now, let the adaptive feedback control law be given by:

(16) $$\begin{array}{c} C_{i}:\;u_{i}\left(t\right)=-{\hat{W}}_{i}{\left(t\right)}^{T}\sigma_{i}\left(x_{si}\left(t\right),c_{i}\left(t\right)\right) \end{array}$$

where $\sigma_{i}\left(x_{si}\left(t\right),c_{i}\left(t\right)\right)=[\beta_{i}^{T}\left(x_{si}\left(t\right)\right)\;,\;x_{si}^{T}\left(t\right)\;,\;c_{i}^{T}\left(t\right){]}^{T}\in R^{g_{i}+n_{i}+m_{i}}$, and ${\hat{W}}_{i}\left(t\right)$ satisfies the weight update law:

(17) $$\begin{array}{c} {\dot{\hat{W}}}_{i}\left(t\right)=\gamma_{i}{\mathrm{Proj}}_{m}[{\hat{W}}_{i}\left(t\right)\;,\;\sigma_{i}\left(x_{si}\left(t\right),c_{i}\left(t\right)\right)e_{si}^{T}\left(t\right)P_{i}B_{i}],\;{\hat{W}}_{i}\left(0\right)={\hat{W}}_{i0} \end{array}$$

with $e_{si}\left(t\right)≜x_{si}\left(t\right)-x_{ri}\left(t\right)\in R^{n_{i}}$ being the error of the triggered module state vector. Note that using Equation (16), Equation (15) can be rewritten as:

(18) $$\begin{array}{cc} {\dot{x}}_{i}\left(t\right)= & A_{ri}x_{i}\left(t\right)+B_{ri}c_{i}\left(t\right)-B_{i}\Lambda_{i}{\tilde{W}}_{i}^{T}\left(t\right)\sigma_{i}\left(x_{si}\left(t\right),c_{i}\left(t\right)\right)-B_{i}\Lambda_{i}g_{i}\left(\cdot \right)+B_{i}\sum_{i∼j}\delta_{ij}\left(x_{j}\left(t\right)\right)\\ & +B_{i}\Lambda_{i}\left(u_{si}\left(t\right)-u_{i}\left(t\right)\right)+B_{i}K_{1i}\left(x_{si}\left(t\right)-x_{i}\left(t\right)\right) \end{array}$$

where $g_{i}\left(\cdot \right)≜W_{i}^{T}\left[\sigma_{i}\left(x_{si}\left(t\right),c_{i}\left(t\right)\right)-\sigma_{i}\left(x_{i}\left(t\right),x_{si}\left(t\right),c_{i}\left(t\right)\right)\right]$, and using Equations (18) and (6), we can write the module error dynamics as:

(19) $$\begin{array}{cc} {\dot{e}}_{i}\left(t\right)= & A_{ri}e_{i}\left(t\right)-B_{i}\Lambda_{i}{\tilde{W}}_{i}^{T}\left(t\right)\sigma_{i}\left(x_{si}\left(t\right),c_{i}\left(t\right)\right)-B_{i}\Lambda_{i}g_{i}\left(\cdot \right)+B_{i}\sum_{i∼j}\delta_{ij}\left(x_{j}\left(t\right)\right)+B_{i}\Lambda_{i}\left(u_{si}\left(t\right)-u_{i}\left(t\right)\right)\\ & +B_{i}K_{1i}\left(x_{si}\left(t\right)-x_{i}\left(t\right)\right) \end{array}$$

The proposed event-triggered decentralized adaptive control algorithm is based on the two-way data exchange structure depicted in Figure 1, where the local controller generates $u_{i}\left(t\right)$ and the uncertain dynamical module is driven by the sampled version of its local control signal $u_{si}\left(t\right)$ depending on an event-triggering mechanism. Similarly, the local controller utilizes $x_{si}\left(t\right)$ that represents the sampled version of the uncertain dynamical module state $x_{i}\left(t\right)$ depending on an event-triggering mechanism. For this purpose, let $\epsilon_{xi}\in R_{+}$ be a given, user-defined sensing threshold to allow for data transmission from the uncertain dynamical system to the controller. In addition, let $\epsilon_{ui}\in R_{+}$ be a given, user-defined actuation threshold to allow for data transmission from the local controller to the uncertain dynamical module. Similar in fashion to [33,35], we now define three logic rules for scheduling the two-way data exchange:

(20) $$\begin{array}{ccc} E_{1i}: & & ∥x_{si}\left(t\right)-x_{i}\left(t\right)∥\leq \epsilon_{xi} \end{array}$$

(21) $$\begin{array}{ccc} E_{2i}: & & ∥u_{si}\left(t\right)-u_{i}\left(t\right)∥\leq \epsilon_{ui} \end{array}$$

(22) $$\begin{array}{ccc} E_{3i}: & & \text{The}\;\text{controller}\;\text{receives}\;x_{si}\left(t\right) \end{array}$$

Specifically, when the inequality in Equation (20) is violated at the $s_{k_{i}}$ moment of the $k_{i}$-th time instant, the uncertain module triggers the measured state signal information, such that $x_{si}\left(t\right)$ is sent to its local controller. Likewise, when Equation (21) is violated or the local controller receives a new transmitted module state from the uncertain dynamical system (i.e., when ${\bar{E}}_{2i}\vee E_{3i}$ is true), then the local controller sends a new control input $u_{si}\left(t\right)$ to the uncertain dynamical module at the $r_{j_{i}}$ moment of the $j_{i}$-th time instant.

We now analyze the system stability and performance of the proposed event-triggered decentralized adaptive control algorithm introduced in this section using the error dynamics given by Equation (19), as well as the data exchange rules $E_{1i}$, $E_{2i}$, and $E_{3i}$ respectively given by Equations (20)–(22). For organizational purposes, the rest of this section, is divided into four subsections. Specifically, we analyze the uniform ultimate boundedness of the resulting closed-loop dynamical system in Section 2.2.1, compute the ultimate bound and highlight the effect of user-defined thresholds and the adaptive controller design parameters on this ultimate bound in Section 2.2.2, show that the proposed architecture does not yield to a Zeno behavior in Section 2.2.3 and generalize the decentralized event-triggered adaptive control algorithm using a state emulator-based framework in Section 2.2.4.

#### 2.2.1. Stability Analysis and Uniform Ultimate Boundedness

We now present the first result of this paper, where the following assumption is needed.

**Assumption 4.**  *$D_{1i}≜\lambda_{\text{min}}\left(R_{i}\right)-2\lambda_{\text{max}}\left(P_{i}\right)∥B_{i}{∥}_{F}\sum_{i∼j}\alpha_{ij}-\sum_{i∼j}\lambda_{\text{max}}\left(P_{j}\right){∥B_{j}∥}_{F}\alpha_{ji}$ is positive by suitable selection of the design parameters.*

**Theorem** **1.**  Consider the uncertain large-scale modular system S consisting of N interconnected modules $S_{i}$ described by Equation (14) subject to Assumptions 1–4. Consider, in addition, the reference model given by Equation (6), and the module feedback control law given by Equations (16) and (17). Moreover, let the data transmission from the uncertain dynamical module to the local controller occur when ${\bar{E}}_{1i}$ is true and the data transmission from the controller to the uncertain dynamical system occur when ${\bar{E}}_{2i}\;\vee \;E_{3i}$ is true. Then, the closed-loop solution $\left(e_{i}\left(t\right),{\tilde{W}}_{i}\left(t\right)\right)$ is uniformly ultimately bounded for all i=1,2,...,N.

**Proof.**  Since the data transmission from the uncertain modules to their local controllers and from the local controllers to the uncertain modules occur when ${\bar{E}}_{1i}$ and ${\bar{E}}_{2i}\vee E_{3i}$ are true, respectively, note that $∥x_{si}\left(t\right)-x_{i}\left(t\right)∥\leq \epsilon_{xi}$ and $∥u_{si}\left(t\right)-u_{i}\left(t\right)∥\leq \epsilon_{ui}$ hold. Consider now the Lyapunov-like function given by:

(23) $$\begin{array}{c} V_{i}\left(e_{i},{\tilde{W}}_{i}\right)=e_{i}^{T}P_{i}e_{i}+\gamma_{i}^{-1}\text{tr}\left({\left({\tilde{W}}_{i}\Lambda_{i}^{\frac{1}{2}}\right)}^{T}\left(\tilde{W_{i}}\Lambda_{i}^{\frac{1}{2}}\right)\right) \end{array}$$

Note that $V_{i}\left(0,0\right)=0$ and $V_{i}\left(e_{i},{\tilde{W}}_{i}\right)>0$ for all $\left(e_{i},{\tilde{W}}_{i}\right)\neq \left(0,0\right)$. The time-derivative of Equation (23) is given by:

(24) $$\begin{array}{c} {\dot{V}}_{i}\left(e_{i}\left(t\right),{\tilde{W}}_{i}\left(t\right)\right)\\ =\;\;2e_{i}^{T}\left(t\right)P{\dot{e}}_{i}\left(t\right)+2\gamma_{i}^{-1}\text{tr}\left({\tilde{W}}_{i}^{T}\left(t\right){\dot{\tilde{W}}}_{i}\left(t\right)\Lambda_{i}\right)\\ \leq \;\;2e_{i}^{T}\left(t\right)P_{i}(A_{ri}e_{i}\left(t\right)-B_{i}\Lambda_{i}{\tilde{W}}_{i}^{T}\left(t\right)\sigma_{i}\left(x_{si}\left(t\right),c_{i}\left(t\right)\right)-B_{i}\Lambda_{i}g_{i}\left(\cdot \right)+B_{i}\sum_{i∼j}\delta_{ij}\left(x_{j}\left(t\right)\right)\\ +B_{i}\Lambda_{i}\left(u_{si}\left(t\right)-u_{i}\left(t\right)\right)+B_{i}K_{1i}\left(x_{si}\left(t\right)-x_{i}\left(t\right)\right))+2\text{tr}\left({\tilde{W}}_{i}^{T}\left(t\right)\Lambda_{i}\sigma_{i}\left(x_{si}\left(t\right),c_{i}\left(t\right)\right)e_{si}^{T}\left(t\right)P_{i}B_{i}\right)\\ \leq \;\;-e_{i}^{T}\left(t\right)R_{i}e_{i}\left(t\right)-2e_{i}^{T}\left(t\right)P_{i}B_{i}\Lambda_{i}g_{i}\left(\cdot \right)+2e_{i}^{T}\left(t\right)P_{i}B_{i}\sum_{i∼j}\delta_{ij}\left(x_{j}\left(t\right)\right)+2e_{i}^{T}\left(t\right)P_{i}B_{i}\Lambda_{i}\left(u_{si}\left(t\right)-u_{i}\left(t\right)\right)\\ +2e_{i}^{T}\left(t\right)P_{i}B_{i}K_{1i}\left(x_{si}\left(t\right)-x_{i}\left(t\right)\right)+2\text{tr}\left({\tilde{W}}_{i}^{T}\left(t\right)\Lambda_{i}\sigma_{i}\left(x_{si}\left(t\right),c_{i}\left(t\right)\right){\left(x_{si}\left(t\right)-x_{i}\left(t\right)\right)}^{T}P_{i}B_{i}\right)\\ \leq \;\;-\lambda_{\text{min}}\left(R_{i}\right)∥e_{i}\left(t\right){∥}^{2}+2∥e_{i}\left(t\right)∥\lambda_{\text{max}}\left(P_{i}\right)∥B_{i}{∥}_{F}∥\Lambda_{i}{∥}_{F}∥g_{i}\left(\cdot \right)∥+∥2e_{i}\left(t\right)P_{i}B_{i}\sum_{i∼j}\delta_{ij}\left(x_{j}\left(t\right)\right)∥\\ +2∥e_{i}\left(t\right)∥\lambda_{\text{max}}\left(P_{i}\right)∥B_{i}{∥}_{F}∥\Lambda_{i}{∥}_{F}\epsilon_{ui}+2∥e_{i}\left(t\right)∥\lambda_{\text{max}}\left(P_{i}\right)∥B_{i}{∥}_{F}∥K_{1i}{∥}_{F}\epsilon_{xi}+2∥{\tilde{W}}_{i}\left(t\right){∥}_{F}{∥\Lambda_{i}∥}_{F}\\ \cdot ∥\sigma_{i}\left(x_{si}\left(t\right),c_{i}\left(t\right)\right)∥\epsilon_{xi}\lambda_{\text{max}}\left(P_{i}\right){∥B_{i}∥}_{F} \end{array}$$

It follows from Assumption 1 that an upper bound for $∥g_{i}\left(\cdot \right)∥$ in Equation (24) can be given by:

(25) $$\begin{array}{cc} ∥g_{i}\left(\cdot \right)∥ & =∥W_{i}^{T}\left[\sigma_{i}\left(x_{si}\left(t\right),c_{i}\left(t\right)\right)-\sigma_{i}\left(x_{i}\left(t\right),x_{si}\left(t\right),c_{i}\left(t\right)\right)\right]∥\\ & \leq \underset{K_{gi}}{\underset{︸}{∥\Lambda_{i}^{-1}{∥}_{F}\omega_{i}^{*}L_{\beta i}}}∥x_{si}\left(t\right)-x_{i}\left(t\right)∥\leq K_{gi}\epsilon_{xi} \end{array}$$

where $K_{gi}\in R_{+}$. In addition, one can compute an upper bound for $∥\sigma_{i}\left(x_{si}\left(t\right),c_{i}\left(t\right)\right)∥$ in Equation (24) as:

(26) $$\begin{array}{cc} ∥\sigma_{i}\left(x_{si}\left(t\right),c_{i}\left(t\right)\right)∥ & \leq ∥\beta_{i}\left(x_{si}\left(t\right)\right)∥+∥x_{si}\left(t\right)∥+∥c_{i}\left(t\right)∥\\ & \leq L_{\beta i}∥x_{si}\left(t\right)∥+∥x_{si}\left(t\right)∥+∥c_{i}\left(t\right)∥\\ & =\left(L_{\beta i}+1\right)\epsilon_{xi}+\left(L_{\beta i}+1\right)∥e_{i}\left(t\right)∥+\left(L_{\beta i}+1\right)x_{ri}^{*}+∥c_{i}\left(t\right)∥ \end{array}$$

where $∥x_{ri}\left(t\right)∥\leq x_{ri}^{*}$. Then, using the bounds given by Equations (25) and (26) in Equation (24), one can write:

(27) $$\begin{array}{c} {\dot{V}}_{i}\left(e_{i}\left(t\right),{\tilde{W}}_{i}\left(t\right)\right)\\ \leq \;\;-\lambda_{\text{min}}\left(R_{i}\right)∥e_{i}\left(t\right){∥}^{2}+(2\lambda_{\text{max}}\left(P_{i}\right)∥B_{i}{∥}_{F}∥\Lambda_{i}{∥}_{F}K_{gi}\epsilon_{xi}+2\lambda_{\text{max}}\left(P_{i}\right)∥B_{i}{∥}_{F}{∥\Lambda_{i}∥}_{F}\epsilon_{ui}+2\lambda_{\text{max}}\left(P_{i}\right)\\ \cdot ∥B_{i}{∥}_{F}∥K_{1i}{∥}_{F}\epsilon_{xi}+2∥{\tilde{W}}_{i}{\left(t\right)∥}_{F}∥\Lambda_{i}{∥}_{F}\left(L_{\beta i}+1\right)\lambda_{\text{max}}\left(P_{i}\right)∥B_{i}{∥}_{F}\epsilon_{xi})∥e_{i}\left(t\right)∥+2∥{\tilde{W}}_{i}\left(t\right){∥}_{F}{∥\Lambda_{i}∥}_{F}\\ \cdot \left(\left(L_{\beta i}+1\right)\epsilon_{xi}+\left(L_{\beta i}+1\right)x_{ri}^{*}+∥c_{i}\left(t\right)∥\right)\lambda_{\text{max}}\left(P_{i}\right)∥B_{i}{∥}_{F}\epsilon_{xi}+∥2e_{i}\left(t\right)P_{i}B_{i}\sum_{i∼j}\delta_{ij}\left(x_{j}\left(t\right)\right)∥\\ =\;\;-c_{1i}∥e_{i}{\left(t\right)∥}^{2}+c_{2i}∥e_{i}\left(t\right)∥+c_{3i}+∥2e_{i}\left(t\right)P_{i}B_{i}\delta_{ij}\left(x_{j}\left(t\right)\right)∥ \end{array}$$

where $c_{1i}≜\lambda_{\text{min}}\left(R_{i}\right)$, $c_{2i}≜2\lambda_{\text{max}}\left(P_{i}\right)∥B_{i}{∥}_{F}∥\Lambda_{i}{∥}_{F}K_{gi}\epsilon_{xi}+2\lambda_{\text{max}}\left(P_{i}\right)∥B_{i}{∥}_{F}∥\Lambda_{i}{∥}_{F}\epsilon_{ui}+2\lambda_{\text{max}}\left(P_{i}\right)∥B_{i}{∥}_{F}{∥K_{1i}∥}_{F}\cdot \epsilon_{xi}$ + 2${\tilde{w}}_{i}^{*}∥\Lambda_{i}{∥}_{F}\left(L_{\beta i}+1\right)\lambda_{\text{max}}\left(P_{i}\right){∥B_{i}∥}_{F}\epsilon_{xi}$ and $c_{3i}≜2{\tilde{w}}_{i}^{*}∥\Lambda_{i}{∥}_{F}\left(\left(L_{\beta i}+1\right)\epsilon_{xi}+\left(L_{\beta i}+1\right)x_{ri}^{*}+∥c_{i}\left(t\right)∥\right)\lambda_{\text{max}}\left(P_{i}\right){∥B_{i}∥}_{F}\epsilon_{xi}$ with $\left|\right|{\tilde{W}}_{i}\left(t\right){\left|\right|}_{F}\leq {\tilde{w}}_{i}^{*}$ due to utilizing the projection operator in the weight update law given by Equation (9).

Since $x_{j}\left(t\right)=e_{j}\left(t\right)+x_{rj}\left(t\right)$ with $∥x_{rj}\left(t\right)∥\leq x_{rj}^{*}$, it follows from Assumption 2 that:

(28) $$\begin{array}{c} ∥\sum_{i∼j}\delta_{ij}\left(x_{j}\left(t\right)\right)∥\leq \sum_{i∼j}\alpha_{ij}\left[∥e_{j}\left(t\right)∥+x_{rj}^{*}\right] \end{array}$$

Furthermore, using Equation (28) in the last term of Equation (27) results in:

(29) $$\begin{array}{cc} ∥2e_{i}\left(t\right)P_{i}B_{i}\delta_{ij}\left(x_{j}\left(t\right)\right)∥ & \leq 2\lambda_{\text{max}}\left(P_{i}\right)∥e_{i}\left(t\right)∥∥B_{i}{∥}_{F}∥\sum_{i∼j}\delta_{ij}\left(x_{j}\left(t\right)\right)∥\\ & \leq 2\lambda_{\text{max}}\left(P_{i}\right)∥e_{i}\left(t\right)∥∥B_{i}{∥}_{F}\sum_{i∼j}\alpha_{ij}\left[∥e_{j}\left(t\right)∥+x_{rj}^{*}\right]\\ & \leq \lambda_{\text{max}}\left(P_{i}\right){∥B_{i}∥}_{F}\sum_{i∼j}\alpha_{ij}\left[2∥e_{i}\left(t\right)∥∥e_{j}\left(t\right)∥+2∥e_{i}\left(t\right)∥x_{rj}^{*}\right]\\ & \leq \lambda_{\text{max}}\left(P_{i}\right){∥B_{i}∥}_{F}\sum_{i∼j}\alpha_{ij}\left[2∥e_{i}{\left(t\right)∥}^{2}+{∥e_{j}\left(t\right)∥}^{2}+{x_{rj}^{*}}^{2}\right] \end{array}$$

where Young’s inequality [46] is considered in the scalar form of $2xy\leq \nu x^{2}+y^{2}/\nu$, where x,y∈R and ν>0, and applied to terms $∥e_{i}\left(t\right)∥∥e_{j}\left(t\right)∥$ and $∥e_{i}\left(t\right)∥x_{rj}^{*}$ with ν=1. Hence, Equation (27) becomes:

(30) $$\begin{array}{cc} {\dot{V}}_{i}\left(e_{i}\left(t\right),{\tilde{W}}_{i}\left(t\right)\right) & \leq \;-\underset{d_{1i}}{\underset{︸}{\left[c_{1i}-2\lambda_{\text{max}}\left(P_{i}\right){∥B_{i}∥}_{F}\sum_{i∼j}\alpha_{ij}\right]}}∥e_{i}{\left(t\right)∥}^{2}+\underset{f_{i}}{\underset{︸}{\lambda_{\text{max}}\left(P_{i}\right){∥B_{i}∥}_{F}}}\sum_{i∼j}\alpha_{ij}{∥e_{j}\left(t\right)∥}^{2}\\ & \;+c_{2i}∥e_{i}\left(t\right)∥+\varphi_{i} \end{array}$$

where $\varphi_{i}≜c_{3i}+\lambda_{\text{max}}\left(P_{i}\right){∥B_{i}∥}_{F}\sum_{i∼j}\alpha_{ij}{x_{rj}^{*}}^{2}$.

Introducing:

(31) $$\begin{array}{c} V\left(\cdot \right)=\sum_{i=1}^{N}V_{i}\left(e_{i}\left(t\right),{\tilde{W}}_{i}\left(t\right)\right) \end{array}$$

for the uncertain system S results in:

(32) $$\begin{array}{cc} \dot{V}\left(\cdot \right) & \leq \;\sum_{i=1}^{N}[-d_{1i}∥e_{i}{\left(t\right)∥}^{2}+f_{i}\sum_{i∼j}\alpha_{ij}∥e_{j}{\left(t\right)∥}^{2}+c_{2i}∥e_{i}\left(t\right)∥+\varphi_{i}]\\ & =\;\sum_{i=1}^{N}[-\underset{D_{1i}}{\underset{︸}{\left(d_{1i}-\sum_{i∼j}f_{j}\alpha_{ji}\right)}}∥e_{i}{\left(t\right)∥}^{2}+c_{2i}∥e_{i}\left(t\right)∥+\varphi_{i}] \end{array}$$

where $D_{1i}>0$ is defined in Assumption 4. Letting $e_{a}\left(t\right)≜{\left[∥e_{1}\left(t\right)∥,\cdots ,∥e_{N}\left(t\right)∥\right]}^{T}$, $D_{1}≜\text{diag}([D_{11},\cdots ,D_{1N}])$, $C_{2}≜\text{diag}\left(\left[c_{21},\cdots ,c_{2N}\right]\right)$ and $\varphi_{a}≜\sum_{i=1}^{N}\varphi_{i}$, Equation (32) can equivalently be written as:

(33) $$\begin{array}{cc} \dot{V}\left(\cdot \right) & \leq -e_{a}^{T}\left(t\right)D_{1}e_{a}\left(t\right)+C_{2}e_{a}\left(t\right)+\varphi_{a}\\ & \leq -\lambda_{\text{min}}\left(D_{1}\right)∥e_{a}\left(t\right){∥}^{2}+\lambda_{\text{max}}\left(C_{2}\right)∥e_{a}\left(t\right)∥+\varphi_{a} \end{array}$$

When $∥e_{a}\left(t\right)∥>\psi$, this renders $\dot{V}\left(\cdot \right)<0$, where $\psi ≜\frac{\frac{\lambda_{\text{max}}\left(C_{2}\right)}{2\sqrt{\lambda_{\text{min}}\left(D_{1}\right)}}+\sqrt{\frac{\lambda_{\text{max}}^{2}\left(C_{2}\right)}{4\lambda_{\text{min}}\left(D_{1}\right)}+\varphi_{a}}}{\sqrt{\lambda_{\text{min}}\left(D_{1}\right)}}$. Hence, $e_{i}\left(t\right)$ and ${\tilde{W}}_{i}\left(t\right)$ are uniformly ultimate bounded for all i=1,2,...,N. ☐

#### 2.2.2. Computation of the Ultimate Bound for System Performance Assessment

For revealing the effect of user-defined thresholds and the event-triggered feedback adaptive controller design parameters to the system performance, the next corollary presents a computation of the ultimate bound for the system S. For this purpose, we define the following, $P_{\min}≜\text{diag}\left(\left[\lambda_{\min}\left(P_{1}\right),\cdots ,\lambda_{\min}\left(P_{N}\right)\right]\right)$, $P_{\max}≜\text{diag}\left(\left[\lambda_{\max}\left(P_{1}\right),\cdots ,\lambda_{\max}\left(P_{N}\right)\right]\right)$, $\gamma_{a}\;≜\;\text{diag}\left(\left[\gamma_{1}^{-1},\cdots ,\gamma_{N}^{-1}\right]\right)$, $\Lambda_{a}≜\text{diag}\left(\left[∥\Lambda_{1}{∥}_{F},\cdots ,{∥\Lambda_{N}∥}_{F}\right]\right)$, ${\tilde{W}}_{a}\left(t\right)≜{\left[∥{\tilde{W}}_{1}{\left(t\right)∥}_{F},\cdots ,{∥{\tilde{W}}_{N}\left(t\right)∥}_{F}\right]}^{T}$.

**Corollary 1.**  *Consider the uncertain dynamical system S consisting of N interconnected modules $S_{i}$ described by Equation (14) subject to Assumptions 1–4. Consider, in addition, the reference model given by Equation (6), and the module feedback control law given by Equations (16) and (17). Moreover, let the data transmission from the uncertain modules to their local controllers occur when ${\bar{E}}_{1i}$ is true and the data transmission from the controllers to the uncertain modules occur when ${\bar{E}}_{2i}\vee E_{3i}$ is true. Then, the ultimate bound of the system error between the uncertain dynamical system and the reference model is given by:*

(34) $$\begin{array}{c} \left|\right|e_{a}\left(t\right)\left|\right|\;\;\leq \;\;\tilde{\Phi }\lambda_{\min}^{-\frac{1}{2}}\left(P_{\min}\right),\;t\geq T \end{array}$$

*where:*

(35) $$\begin{array}{c} \tilde{\Phi }≜{\left[\lambda_{\max}\left(P_{\max}\right)\psi^{2}+\lambda_{\max}\left(\gamma_{a}\right)\lambda_{\max}\left(\Lambda_{a}\right){∥{\tilde{W}}_{a}\left(t\right)∥}^{2}\right]}^{\frac{1}{2}}\;\;\;\; \end{array}$$

**Proof.**  It follows from the proof of Theorem 1 that $\dot{V}\left(e_{a}\left(t\right),{\tilde{W}}_{a}\left(t\right)\right)\leq 0$ outside the compact set given by:

(36) $$\begin{array}{c} S≜\left\{e_{a}\left(t\right):∥e_{a}\left(t\right)∥\leq \psi \right\} \end{array}$$

That is, since $V(e_{a}\left(t\right),{\tilde{W}}_{a}\left(t\right))$ cannot grow outside S, the evolution of $V(e_{a}\left(t\right),{\tilde{W}}_{a}\left(t\right))$ is upper bounded by:

(37) $$\begin{array}{ccc} V(e_{a}\left(t\right),{\tilde{W}}_{a}\left(t\right)) & \leq & \max_{e_{a}\left(t\right)\in S}V\left(e_{a}\left(t\right),{\tilde{W}}_{a}\left(t\right)\right)\\ & = & \lambda_{\max}\left(P_{\max}\right)\psi^{2}+\lambda_{\max}\left(\gamma_{a}\right)\lambda_{\max}\left(\Lambda_{a}\right){∥{\tilde{W}}_{a}\left(t\right)∥}^{2}\\ & = & {\tilde{\Phi }}^{2} \end{array}$$

It follows from $e_{a}^{T}P_{\min}e_{a}\leq V\left(e_{a},{\tilde{W}}_{a}\right)$ that ${∥e_{a}\left(t\right)∥}^{2}\leq \frac{{\tilde{\Phi }}^{2}}{\lambda_{\min}\left(P_{\min}\right)}$, and Equation (34) is immediate. ☐

#### 2.2.3. Computation of the Event-Triggered Inter-Sample Time Lower Bound

We now show that the proposed event-triggered decentralized adaptive control architecture does not yield to a Zeno behavior, which implies that it does not require a continuous two-way data exchange and reduces wireless network utilization. For the following corollary presenting the result of this subsection, we consider $r_{q_{i}}^{k_{i}}\in \left(s_{k_{i}},s_{k_{i}+1}\right)$ to be the $q_{i}$-th time instant when $E_{2i}$ is violated over $\left(s_{k_{i}},s_{k_{i}+1}\right)$, and since ${\left\{s_{k_{i}}\right\}}_{k_{i}=1}^{\infty }$ is a subsequence of ${\left\{r_{j_{i}}\right\}}_{j_{i}=1}^{\infty }$, it follows that ${\left\{r_{j_{i}}\right\}}_{j_{i}=1}^{\infty }\;=\;{\left\{s_{k_{i}}\right\}}_{k_{i}=1}^{\infty }⋃{\left\{r_{q_{i}}^{k_{i}}\right\}}_{k_{i}=1,q_{i}=1}^{\infty ,m_{k_{i}}}$, where $m_{k_{i}}\in N$ is the number of violation times of $E_{2i}$ over $\left(s_{k_{i}},s_{k_{i}+1}\right)$.

**Corollary 2.**  *Consider the uncertain dynamical system S consisting of N interconnected modules $S_{i}$ described by Equation (14) subject to Assumptions 1–4. Consider, in addition, the reference model given by Equation (6), and the module feedback control law given by Equations (16) and (17). Moreover, let the data transmission from the uncertain dynamical module to the local controller occur when ${\bar{E}}_{1i}$ is true and the data transmission from the controller to the uncertain dynamical system occur when ${\bar{E}}_{2i}\vee E_{3i}$ is true. Then, there exist positive scalars $\alpha_{xi}≜\frac{\epsilon_{xi}}{\Phi_{1i}}$ and $\alpha_{ui}≜\frac{\epsilon_{ui}}{\Phi_{2i}}$ such that:*

(38) $$\begin{array}{ccc} s_{k_{i}+1}-s_{k_{i}} & > & \alpha_{xi},\;\forall k_{i}\in N \end{array}$$

(39) $$\begin{array}{ccc} r_{q_{i}+1}^{k_{i}}-r_{q_{i}}^{k_{i}} & > & \alpha_{ui},\;\forall q_{i}\in \left\{0,...,m_{k_{i}}\right\},\;\forall k_{i}\in N \end{array}$$

**Proof.**  The time derivative of $∥x_{si}\left(t\right)-x_{i}\left(t\right)∥$ over $t\in \left(s_{k_{i}},s_{k_{i}+1}\right)$, $\forall k_{i}\in N$, is given by:

(40) $$\begin{array}{ccc} \frac{d}{dt}∥x_{si}\left(t\right)-x_{i}\left(t\right)∥ & \leq & ∥{\dot{x}}_{si}\left(t\right)-{\dot{x}}_{i}\left(t\right)∥=∥{\dot{x}}_{i}\left(t\right)∥\\ & \leq & ∥A_{ri}{∥}_{F}\left[∥e_{i}\left(t\right)∥+x_{ri}^{*}\right]+∥B_{ri}{∥}_{F}∥c_{i}\left(t\right)∥+∥B_{i}{∥}_{F}∥\Lambda_{i}{∥}_{F}{\tilde{w}}_{i}^{*}[L_{\beta i}(\epsilon_{xi}+∥e_{i}\left(t\right)∥\\ & & +x_{ri}^{*})+∥K_{1i}{∥}_{F}\left(\epsilon_{xi}+∥e_{i}\left(t\right)∥+x_{ri}^{*}\right)+∥K_{2i}{∥}_{F}∥c_{i}\left(t\right)∥]+∥B_{i}{∥}_{F}{∥\Lambda_{i}∥}_{F}K_{gi}\epsilon_{xi}\\ & & +∥B_{i}{∥}_{F}\sum_{i∼j}\alpha_{ij}\left(∥e_{j}\left(t\right)∥+x_{rj}^{*}\right)+∥B_{i}{∥}_{F}∥\Lambda_{i}{∥}_{F}\epsilon_{ui}+∥B_{i}{∥}_{F}{∥K_{1i}∥}_{F}\epsilon_{xi} \end{array}$$

Since the closed-loop dynamical system is uniformly ultimately bounded by Theorem 1, there exists an upper bound to Equation (40). Letting $\Phi_{1i}$ denote this upper bound and with the initial condition satisfying $\lim_{t\to s_{k_{i}}^{+}}∥x_{si}\left(t\right)-x_{i}\left(t\right)∥=0$, it follows from Equation (40) that:

(41) $$\begin{array}{c} ∥x_{si}\left(t\right)-x_{i}\left(t\right)∥\leq \Phi_{1i}\left(t-s_{k_{i}}\right),\;\forall t\in \left(s_{k_{i}},s_{k_{i}+1}\right)\;\; \end{array}$$

Therefore, when ${\bar{E}}_{1i}$ is true, then $\lim_{t\to s_{k_{i}+1}^{-}}∥x_{si}\left(t\right)-x_{i}\left(t\right)∥=\epsilon_{xi}$, and it then follows from Equation (41) that $s_{k_{i}+1}-s_{k_{i}}\geq \alpha_{xi}$.

Similarly, the time derivative of $∥u_{si}\left(t\right)-u_{i}\left(t\right)∥$ over $t\in \left(r_{q_{i}}^{k_{i}},r_{q_{i}+1}^{k_{i}}\right),\forall q_{i}\in N$, is given by:

(42) $$\begin{array}{ccc} \frac{d}{dt}∥u_{si}\left(t\right)-u_{i}\left(t\right)∥ & \leq & ∥{\dot{u}}_{si}\left(t\right)-{\dot{u}}_{i}\left(t\right)∥=∥{\dot{u}}_{i}\left(t\right)∥\\ & = & ∥{\dot{\hat{W}}}_{i}^{T}\left(t\right)\sigma_{i}\left(x_{si}\left(t\right),c_{i}\left(t\right)\right)+{\hat{W}}_{i}^{T}\left(t\right){\dot{\sigma }}_{i}\left(x_{si}\left(t\right),c_{i}\left(t\right)\right)∥\\ & \leq & \gamma_{i}{∥B_{i}∥}_{F}\lambda_{\text{max}}\left(P_{i}\right)∥e_{si}\left(t\right)∥{∥\sigma_{i}\left(x_{si}\left(t\right),c_{i}\left(t\right)\right)∥}^{2}+∥\Lambda_{i}^{-1}{∥}_{F}∥K_{2i}{∥}_{F}∥{\dot{c}}_{i}\left(t\right)∥\\ & \leq & \gamma_{i}{∥B_{i}∥}_{F}\lambda_{\text{max}}\left(P_{i}\right)\left(∥e_{i}\left(t\right)∥+\epsilon_{xi}\right)[L_{\beta i}\left(\epsilon_{xi}+∥e_{i}\left(t\right)∥+x_{ri}^{*}\right)+∥K_{1i}{∥}_{F}(\epsilon_{xi}\\ & & +∥e_{i}\left(t\right)∥+x_{ri}^{*})+∥K_{2i}{∥}_{F}∥c_{i}\left(t\right)∥{]}^{2}+∥\Lambda_{i}^{-1}{∥}_{F}∥K_{2i}{∥}_{F}∥{\dot{c}}_{i}\left(t\right)∥ \end{array}$$

Once again, since the closed-loop dynamical system is uniformly ultimately bounded by Theorem 1, there exists an upper bound to Equation (42). Letting $\Phi_{2i}$ denote this upper bound, and with the initial condition satisfying $\lim_{t\to r_{q_{i}}^{k_{i+}}}∥u_{si}\left(t\right)-u_{i}\left(t\right)∥=0$, it follows from Equation (42) that:

(43) $$\begin{array}{c} ∥u_{si}\left(t\right)-u_{i}\left(t\right)∥\leq \Phi_{2i}\left(t-r_{q_{i}}^{k_{i}}\right),\;\forall t\in \left(r_{q_{i}}^{k_{i}},r_{q_{i}+1}^{k_{i}}\right)\;\; \end{array}$$

Therefore, when ${\bar{E}}_{2i}\vee E_{3i}$ is true, then $\lim_{t\to r_{q_{i}+1}^{k_{i-}}}∥u_{si}\left(t\right)-u_{i}\left(t\right)∥=\epsilon_{ui}$, and it then follows from Equation (43) that $r_{q_{i}+1}^{k_{i}}-r_{q_{i}}^{k_{i}}\geq \alpha_{ui}$. ☐

Corollary 2 shows that the inter-sample times for the module state vector and decentralized feedback control vector are bounded away from zero, and hence, the proposed event-triggered adaptive control approach does not yield to a Zeno behavior. As discussed earlier, this implies that the proposed event-triggered decentralized adaptive control methodology does not require a continuous two-way data exchange, and it reduces wireless network utilization.

#### 2.2.4. Generalizations to the Event-Triggered Decentralized Adaptive Control with State Emulator

We now generalize our framework to a state emulator-based design, since this framework has the capability to suppress possible high-frequency oscillation in the control signal of the uncertain module $S_{i}$ [10,13,37,38,39,40,41,42]. Consider the (modified) reference system, so-called the state emulator of $S_{i}$, given by:

(44) $$\begin{array}{c} {\dot{\hat{x}}}_{i}\left(t\right)=A_{ri}{\hat{x}}_{i}\left(t\right)+B_{ri}c_{i}\left(t\right)+L_{i}\left(x_{si}\left(t\right)-{\hat{x}}_{i}\left(t\right)\right),\;{\hat{x}}_{i}\left(0\right)={\hat{x}}_{i0}\;\; \end{array}$$

where $L_{i}\in R_{+}^{n_{i}\times n_{i}}\cap D^{n_{i}\times n_{i}}$ is the state emulator gain. Letting ${\hat{e}}_{i}\left(t\right)≜{\hat{x}}_{i}\left(t\right)-x_{ri}\left(t\right)\in R^{n_{i}}$, the reference model error dynamics capturing the difference between the ideal reference model in Equation (6) and the state emulator-based (modified) reference model in Equation (44) is given by:

(45) $$\begin{array}{c} {\dot{\hat{e}}}_{i}\left(t\right)=A_{ri}{\hat{e}}_{i}\left(t\right)+L_{i}\left(x_{si}\left(t\right)-{\hat{x}}_{i}\left(t\right)\right)\;\; \end{array}$$

In addition, letting ${\tilde{x}}_{i}\left(t\right)≜x_{i}\left(t\right)-{\hat{x}}_{i}\left(t\right)\in R^{n_{i}}$ to denote the system state error vector, the (state emulator-based) system error dynamics follows from Equations (18) and (44) as:

(46) $$\begin{array}{ccc} {\dot{\tilde{x}}}_{i}\left(t\right) & = & A_{Li}{\tilde{x}}_{i}\left(t\right)-B_{i}\Lambda_{i}{\tilde{W}}_{i}^{T}\left(t\right)\sigma_{i}\left(x_{si}\left(t\right),c_{i}\left(t\right)\right)-B_{i}\Lambda_{i}g_{i}\left(\cdot \right)+B_{i}\delta_{ij}\left(x_{j}\left(t\right)\right)+B_{i}\Lambda_{i}\left(u_{si}\left(t\right)-u_{i}\left(t\right)\right)\\ & & +\left(B_{i}K_{1i}-L_{i}\right)\left(x_{si}\left(t\right)-x_{i}\left(t\right)\right),\;{\tilde{x}}_{i}\left(0\right)={\tilde{x}}_{i0}\;\; \end{array}$$

where $A_{Li}≜A_{ri}-L_{i}\in R^{n_{i}\times n_{i}}$ is Hurwitz by a suitable selection of the state emulator gain $L_{i}$ (e.g., $A_{Li}$ is Hurwitz with $L_{i}=\kappa_{i}I$, $\kappa_{i}\in R_{+}$, since $A_{ri}$ is Hurwitz). To maintain system stability, we utilize the adaptive controller given by Equation (16) with the update law described by:

(47) $$\begin{array}{c} {\dot{\hat{W}}}_{i}\left(t\right)≜\gamma_{i}{\mathrm{Proj}}_{m}[{\hat{W}}_{i}\left(t\right)\;,\;\sigma_{i}\left(x_{si}\left(t\right),c_{i}\left(t\right)\right){\left(x_{si}\left(t\right)-{\hat{x}}_{i}\left(t\right)\right)}^{T}P_{i}B_{i}],\;{\hat{W}}_{i}\left(0\right)={\hat{W}}_{i0} \end{array}$$

where $P_{i}\in R_{+}^{n_{i}\times n_{i}\cap S^{n_{i}\times n_{i}}}$ is the unique solution of the algebraic Riccati equation:

(48) $$\begin{array}{c} 0=A_{Li}^{T}P_{i}+P_{i}A_{Li}-P_{i}B_{i}R_{i}^{-1}B_{i}^{T}P_{i}+Q_{i} \end{array}$$

with $R_{i}\in R_{+}^{m_{i}\times m_{i}}\cap S^{n_{i}\times n_{i}}$ and $Q_{i}\in R_{+}^{n_{i}\times n_{i}}\cap S^{n_{i}\times n_{i}}$.

Note from [10,42] that the state emulator-based adaptive control framework achieves stringent transient and steady-state system performance specifications by judiciously choosing the learning rate $\gamma_{i}$ and the state emulator gain $L_{i}$ without causing high-frequency oscillations in the controller response, unlike standard model reference adaptive controllers overviewed earlier in this section. We also note that if one selects $L_{i}=0$, then the results of this paper hold for standard model reference adaptive controllers, and hence, there is no loss in generality in using a state emulator-based adaptive control framework for the main results of this paper.

Consider a parameter-dependent Riccati equation [23,47] given by:

(49) $$\begin{array}{ccc} 0 & = & A_{ri}^{T}{\tilde{P}}_{i}+{\tilde{P}}_{i}A_{ri}+{\tilde{Q}}_{i} \end{array}$$

(50) $$\begin{array}{ccc} {\tilde{Q}}_{i} & = & \mu_{i}{\tilde{P}}_{i}L_{i}L_{i}^{T}{\tilde{P}}_{i}+{\tilde{Q}}_{oi}, \end{array}$$

where ${\tilde{P}}_{i}\in R_{+}^{n_{i}\times n_{i}}$ is a unique solution with ${\tilde{Q}}_{oi}\in R_{+}^{n_{i}\times n_{i}}$ and $\mu_{i}>0$.

**Remark 1 [23].**  *Let $0<\mu_{i}<{\bar{\mu }}_{i}$ define the largest set within which there is a positive-definite solution for ${\tilde{P}}_{i}$. Since ${\tilde{P}}_{i}>0$ for $\mu_{i}=0$ and ${\tilde{P}}_{i}>0$ depends continuously on $\mu_{i}$, the existence of ${\tilde{P}}_{i}\left(\mu_{i}\right)>0$ for $0<\mu_{i}<{\bar{\mu }}_{i}$ is assured.*

The next lemma shows that for $\mu_{i}<{\bar{\mu }}_{i}$, Equations (49) and (50) can reliably be solved for ${\tilde{P}}_{i}>0$ using the Potter approach given in [48]. This also implies that ${\bar{\mu }}_{i}$ can be determined by searching for the boundary value, ${\bar{\mu }}_{i}$. We employ notation ric(·) and dom(·) as defined in [48].

**Lemma 1 [23,48].**  *Let ${\tilde{P}}_{i}>0$ satisfy the parameter dependent Riccati equation given by Equations (49) and (50), and let the modified Hamiltonian be given by:*

(51) $$\begin{array}{c} H_{i}=\left[\begin{array}{cc} A_{ri} & \mu_{i}L_{i}L_{i}^{T}\\ -{\tilde{Q}}_{oi} & -A_{ri}^{T} \end{array}\right] \end{array}$$

*Then, for all $0<\mu_{i}<{\bar{\mu }}_{i}$, $H_{i}\in \text{dom}\left(\text{ric}\right)$ and ${\tilde{P}}_{i}=\text{ric}\left(H_{i}\right)$.*

**Assumption 5.**  *$D_{1i}≜\lambda_{\text{min}}\left(Q_{i}\right)-\lambda_{\text{min}}\left(R_{i}^{-1}\right)\lambda_{\text{max}}^{2}\left(P_{i}\right)∥B_{i}{∥}_{F}^{2}-\frac{l_{i}}{\mu_{i}}-3\lambda_{\text{max}}\left(P_{i}\right)∥B_{i}{∥}_{F}\sum_{i∼j}\alpha_{ij}-\sum_{i∼j}\lambda_{\text{max}}\left(P_{j}\right){∥B_{j}∥}_{F}\alpha_{ji}$ and $D_{2i}≜l_{i}\lambda_{\text{min}}\left({\tilde{Q}}_{oi}\right)-\sum_{i∼j}\lambda_{\text{max}}\left(P_{j}\right){∥B_{j}∥}_{F}\alpha_{ji}$, $l_{i}>0$, are positive by suitable selection of the design parameters.*

**Corollary 3.**  *Consider the uncertain dynamical system S consisting of N interconnected modules $S_{i}$ described by Equation (14) subject to Assumptions 1–3 and 5. Consider in addition, the ideal reference model given by Equation (6), the state emulator given by Equation (44) and the module feedback control law given by Equations (16) and (47). Moreover, let the data transmission from the uncertain dynamical module to the local controller occur when ${\bar{E}}_{1i}$ is true and the data transmission from the controller to the uncertain dynamical system occur when ${\bar{E}}_{2i}\vee E_{3i}$ is true. Then, the closed-loop solution $\left({\tilde{x}}_{i}\left(t\right),{\tilde{W}}_{i}\left(t\right),{\hat{e}}_{i}\left(t\right)\right)$ is uniformly ultimately bounded for all i=1,2,...,N.*

**Proof.**  Consider the Lyapunov-like function given by:

(52) $$\begin{array}{c} V_{i}\left({\tilde{x}}_{i},{\tilde{W}}_{i},{\hat{e}}_{i}\right)={\tilde{x}}_{i}^{T}P_{i}{\tilde{x}}_{i}+\gamma_{i}^{-1}\mathrm{tr}{\left({\tilde{W}}_{i}\Lambda_{i}^{\frac{1}{2}}\right)}^{T}\left({\tilde{W}}_{i}\Lambda_{i}^{\frac{1}{2}}\right)+l_{i}{\hat{e}}_{i}^{T}{\tilde{P}}_{i}{\hat{e}}_{i} \end{array}$$

where $l_{i}>0$ and ${\tilde{P}}_{i}>0$ satisfies the parameter dependent Riccati equation in Equations (49) and (50). Note that $V_{i}\left(0,0,0\right)=0$ and $V_{i}\left({\tilde{x}}_{i},{\tilde{W}}_{i},{\hat{e}}_{i}\right)>0$ for all $\left({\tilde{x}}_{i},{\tilde{W}}_{i},{\hat{e}}_{i}\right)\neq \left(0,0,0\right)$. The time-derivative of Equation (52) is given by:

(53) $$\begin{array}{ccc} & & {\dot{V}}_{i}\left({\tilde{x}}_{i}\left(t\right),{\tilde{W}}_{i}\left(t\right),{\hat{e}}_{i}\left(t\right)\right)\\ & & =\;2{\tilde{x}}_{i}^{T}\left(t\right)P_{i}{\dot{\tilde{x}}}_{i}\left(t\right)+2\gamma_{i}^{-1}\mathrm{tr}{\left({\tilde{W}}_{i}\left(t\right)\Lambda_{i}^{\frac{1}{2}}\right)}^{T}\left({\dot{\tilde{W}}}_{i}\left(t\right)\Lambda_{i}^{\frac{1}{2}}\right)+2l_{i}{\hat{e}}_{i}^{T}\left(t\right){\tilde{P}}_{i}{\dot{\hat{e}}}_{i}\left(t\right)\\ & & \leq \;2{\tilde{x}}_{i}^{T}\left(t\right)P_{i}[A_{L_{i}}{\tilde{x}}_{i}\left(t\right)-B_{i}\Lambda_{i}{\tilde{W}}_{i}^{T}\left(t\right)\sigma_{i}\left(x_{si}\left(t\right),c_{i}\left(t\right)\right)-B_{i}\Lambda_{i}g_{i}\left(\cdot \right)+B_{i}\delta_{ij}\left(x_{j}\left(t\right)\right)+B_{i}\Lambda_{i}\left(u_{si}\left(t\right)-u_{i}\left(t\right)\right)\\ & & +\left(B_{i}K_{1i}-L_{i}\right)\left(x_{si}\left(t\right)-x_{i}\left(t\right)\right)]+2\mathrm{tr}{\tilde{W}}_{i}^{T}\left(t\right)\sigma_{i}\left(x_{si}\left(t\right),c_{i}\left(t\right)\right){\left(x_{si}\left(t\right)-{\hat{x}}_{i}\left(t\right)\right)}^{T}P_{i}B_{i}\Lambda_{i}\\ & & +2l_{i}{\hat{e}}_{i}^{T}\left(t\right){\tilde{P}}_{i}\left[A_{ri}{\hat{e}}_{i}\left(t\right)+L_{i}\left(x_{si}\left(t\right)-{\hat{x}}_{i}\left(t\right)\right)\right]\\ & & \leq \;-{\tilde{x}}_{i}^{T}\left(t\right)Q_{i}{\tilde{x}}_{i}\left(t\right)+{\tilde{x}}_{i}^{T}\left(t\right)P_{i}B_{i}R_{i}^{-1}B_{i}^{T}P_{i}{\tilde{x}}_{i}\left(t\right)-2{\tilde{x}}_{i}^{T}\left(t\right)P_{i}B_{i}\Lambda_{i}g_{i}\left(\cdot \right)+2{\tilde{x}}_{i}^{T}\left(t\right)P_{i}B_{i}\delta_{ij}\left(x_{j}\left(t\right)\right)\\ & & +2{\tilde{x}}_{i}^{T}\left(t\right)P_{i}B_{i}\Lambda_{i}\left(u_{si}\left(t\right)-u_{i}\left(t\right)\right)+2{\tilde{x}}_{i}^{T}\left(t\right)P_{i}\left(B_{i}K_{1i}-L_{i}\right)\left(x_{si}\left(t\right)-x_{i}\left(t\right)\right)+2\mathrm{tr}{\tilde{W}}_{i}{\left(t\right)}^{T}\\ & & \cdot \sigma_{i}\left(x_{si}\left(t\right),c_{i}\left(t\right)\right){\left(x_{si}\left(t\right)-x_{i}\left(t\right)\right)}^{T}P_{i}B_{i}\Lambda_{i}-l_{i}{\hat{e}}_{i}^{T}\left(t\right){\tilde{Q}}_{oi}{\hat{e}}_{i}\left(t\right)-l_{i}{\hat{e}}_{i}^{T}\left(t\right)\mu_{i}{\tilde{P}}_{i}L_{i}L_{i}^{T}{\tilde{P}}_{i}{\hat{e}}_{i}\left(t\right)\\ & & +2l_{i}{\hat{e}}_{i}^{T}\left(t\right){\tilde{P}}_{i}L_{i}\left(x_{si}\left(t\right)-x_{i}\left(t\right)\right)+2l_{i}{\hat{e}}_{i}^{T}\left(t\right){\tilde{P}}_{i}L_{i}{\tilde{x}}_{i}\left(t\right) \end{array}$$

Young’s inequality [46] applied to the last term in Equation (53) produces:

(54) $$\begin{array}{c} 2l_{i}{\hat{e}}_{i}^{T}\left(t\right){\tilde{P}}_{i}L_{i}{\tilde{x}}_{i}\left(t\right)\leq \mu_{i}l_{i}{\hat{e}}_{i}^{T}\left(t\right){\tilde{P}}_{i}L_{i}L_{i}^{T}{\tilde{P}}_{i}{\hat{e}}_{i}\left(t\right)+\frac{l_{i}}{\mu_{i}}{\tilde{x}}_{i}^{T}\left(t\right){\tilde{x}}_{i}\left(t\right) \end{array}$$

Using Equation (54) in Equation (53) yields:

(55) $$\begin{array}{ccc} & & {\dot{V}}_{i}\left({\tilde{x}}_{i}\left(t\right),{\tilde{W}}_{i}\left(t\right),{\hat{e}}_{i}\left(t\right)\right)\\ & & \leq \;-{\tilde{x}}_{i}^{T}\left(t\right)Q_{i}{\tilde{x}}_{i}\left(t\right)+{\tilde{x}}_{i}^{T}\left(t\right)P_{i}B_{i}R_{i}^{-1}B_{i}^{T}P_{i}{\tilde{x}}_{i}\left(t\right)-2{\tilde{x}}_{i}^{T}\left(t\right)P_{i}B_{i}\Lambda_{i}g_{i}\left(\cdot \right)+2{\tilde{x}}_{i}^{T}\left(t\right)P_{i}B_{i}\delta_{ij}\left(x_{j}\left(t\right)\right)\\ & & +2{\tilde{x}}_{i}^{T}\left(t\right)P_{i}B_{i}\Lambda_{i}\left(u_{si}\left(t\right)-u_{i}\left(t\right)\right)+2{\tilde{x}}_{i}^{T}\left(t\right)P_{i}\left(B_{i}K_{1i}-L_{i}\right)\left(x_{si}\left(t\right)-x_{i}\left(t\right)\right)+2\mathrm{tr}{\tilde{W}}_{i}^{T}\left(t\right)\sigma_{i}\left(x_{si}\left(t\right),c_{i}\left(t\right)\right)\\ & & \cdot {\left(x_{si}\left(t\right)-x_{i}\left(t\right)\right)}^{T}P_{i}B_{i}\Lambda_{i}-l_{i}{\hat{e}}_{i}^{T}\left(t\right){\tilde{Q}}_{oi}{\hat{e}}_{i}\left(t\right)+2l_{i}{\hat{e}}_{i}^{T}\left(t\right){\tilde{P}}_{i}L_{i}\left(x_{si}\left(t\right)-x_{i}\left(t\right)\right)+\frac{l_{i}}{\mu_{i}}{\tilde{x}}_{i}^{T}\left(t\right){\tilde{x}}_{i}\left(t\right)\\ & & \leq \;-\lambda_{\text{min}}\left(R_{i}\right)∥{\tilde{x}}_{i}\left(t\right){∥}^{2}+\lambda_{\text{min}}\left(R_{i}^{-1}\right)\lambda_{\text{max}}^{2}\left(P_{i}\right)∥B_{i}{∥}_{F}^{2}∥{\tilde{x}}_{i}\left(t\right){∥}^{2}+2\lambda_{\text{max}}\left(P_{i}\right)∥B_{i}{∥}_{F}∥\Lambda_{i}{∥}_{F}∥g_{i}\left(\cdot \right)∥∥{\tilde{x}}_{i}\left(t\right)∥\\ & & +∥2{\tilde{x}}_{i}\left(t\right)P_{i}B_{i}\delta_{ij}\left(x_{j}\left(t\right)\right)∥+2∥{\tilde{x}}_{i}\left(t\right)∥\lambda_{\text{max}}\left(P_{i}\right)∥B_{i}{∥}_{F}∥\Lambda_{i}{∥}_{F}\epsilon_{ui}+2∥{\tilde{x}}_{i}\left(t\right)∥\lambda_{\text{max}}\left(P_{i}\right)({∥B_{i}K_{1i}∥}_{F}\\ & & +∥L_{i}{∥}_{F})\epsilon_{xi}+2∥{\tilde{W}}_{i}{\left(t\right)∥}_{F}∥\sigma_{i}\left(x_{si}\left(t\right),c_{i}\left(t\right)\right)∥\epsilon_{xi}\lambda_{\text{max}}\left(P_{i}\right)∥B_{i}{∥}_{F}∥\Lambda_{i}{∥}_{F}-l_{i}\lambda_{\text{min}}\left({\tilde{Q}}_{oi}\right){∥{\hat{e}}_{i}\left(t\right)∥}^{2}\\ & & +2l_{i}∥{\hat{e}}_{i}\left(t\right)∥\lambda_{\text{max}}\left({\tilde{P}}_{i}\right)∥L_{i}{∥}_{F}\epsilon_{xi}+\frac{l_{i}}{\mu_{i}}{∥{\tilde{x}}_{i}\left(t\right)∥}^{2} \end{array}$$

Using Equations (25) and (26), Equation (55) can be written:

(56) $$\begin{array}{cc} & {\dot{V}}_{i}\left({\tilde{x}}_{i}\left(t\right),{\tilde{W}}_{i}\left(t\right),{\hat{e}}_{i}\left(t\right)\right)\\ & \leq \;\;-\left(\lambda_{\text{min}}\left(Q_{i}\right)-\lambda_{\text{min}}\left(R_{i}^{-1}\right)\lambda_{\text{max}}^{2}\left(P_{i}\right){∥B_{i}∥}_{F}^{2}-\frac{l_{i}}{\mu_{i}}\right)∥{\tilde{x}}_{i}{\left(t\right)∥}^{2}-l_{i}\lambda_{\text{min}}\left({\tilde{Q}}_{oi}\right){∥{\hat{e}}_{i}\left(t\right)∥}^{2}+(2\lambda_{\text{max}}\left(P_{i}\right)\\ & \cdot ∥B_{i}{∥}_{F}∥\Lambda_{i}{∥}_{F}K_{gi}\epsilon_{xi}+2\lambda_{\text{max}}\left(P_{i}\right)∥B_{i}{∥}_{F}∥\Lambda_{i}{∥}_{F}\epsilon_{ui}+2\lambda_{\text{max}}\left(P_{i}\right)∥B_{i}{∥}_{F}∥K_{1i}{∥}_{F}\epsilon_{xi}+2∥{\tilde{W}}_{i}\left(t\right){∥}_{F}{∥\Lambda_{i}∥}_{F}\\ & \cdot \left(L_{\beta i}+1\right)\lambda_{\text{max}}\left(P_{i}\right)∥B_{i}{∥}_{F}\epsilon_{xi})∥{\tilde{x}}_{i}\left(t\right)∥+2∥{\tilde{W}}_{i}\left(t\right){∥}_{F}{∥\Lambda_{i}∥}_{F}\left(\left(L_{\beta i}+1\right)\epsilon_{xi}+\left(L_{\beta i}+1\right)x_{ri}^{*}+∥c_{i}\left(t\right)∥\right)\\ & \cdot \lambda_{\text{max}}\left(P_{i}\right)∥B_{i}{∥}_{F}\epsilon_{xi}+∥2{\tilde{x}}_{i}\left(t\right)P_{i}B_{i}\delta_{ij}\left(x_{j}\left(t\right)\right)∥+2l_{i}\lambda_{\text{max}}\left({\tilde{P}}_{i}\right)∥L_{i}{∥}_{F}\epsilon_{xi}∥{\hat{e}}_{i}\left(t\right)∥\\ & =\;\;-c_{1i}∥{\tilde{x}}_{i}{\left(t\right)∥}^{2}-c_{2i}∥{\hat{e}}_{i}{\left(t\right)∥}^{2}+c_{3i}∥{\tilde{x}}_{i}\left(t\right)∥+c_{4i}∥{\hat{e}}_{i}\left(t\right)∥+c_{5i}+∥2{\tilde{x}}_{i}\left(t\right)P_{i}B_{i}\delta_{ij}\left(x_{j}\left(t\right)\right)∥ \end{array}$$

where $c_{1i}\;≜\;\lambda_{\text{min}}\left(Q_{i}\right)-\lambda_{\text{min}}\left(R_{i}^{-1}\right)\lambda_{\text{max}}^{2}\left(P_{i}\right){∥B_{i}∥}_{F}^{2}-\frac{l_{i}}{\mu_{i}}$, $\;c_{2i}\;≜\;l_{i}\lambda_{\text{min}}\left({\tilde{Q}}_{oi}\right)$, $c_{3i}≜2\lambda_{\text{max}}\left(P_{i}\right)∥B_{i}{∥}_{F}{∥\Lambda_{i}∥}_{F}K_{gi}\epsilon_{xi}+2\lambda_{\text{max}}\left(P_{i}\right)∥B_{i}{∥}_{F}∥\Lambda_{i}{∥}_{F}\epsilon_{ui}+2\lambda_{\text{max}}\left(P_{i}\right)∥B_{i}{∥}_{F}∥K_{1i}{∥}_{F}\epsilon_{xi}+2{\tilde{w}}_{i}^{*}∥\Lambda_{i}∥\left(L_{\beta i}+1\right)\lambda_{\text{max}}\left(P_{i}\right){∥B_{i}∥}_{F}\epsilon_{xi},\;c_{4i}≜\;2l_{i}\lambda_{\text{max}}\left({\tilde{P}}_{i}\right)∥L_{i}{∥}_{F}\epsilon_{xi}$ and $\;c_{5i}\;≜\;2{\tilde{w}}_{i}^{*}∥\Lambda_{i}{∥}_{F}\left(\left(L_{\beta i}+1\right)\epsilon_{xi}+\left(L_{\beta i}+1\right)x_{ri}^{*}+∥c_{i}\left(t\right)∥\right)\lambda_{\text{max}}\left(P_{i}\right){∥B_{i}∥}_{F}\epsilon_{xi}$.

Since $x_{j}\left(t\right)={\tilde{x}}_{j}\left(t\right)+{\hat{e}}_{j}\left(t\right)+x_{rj}\left(t\right)$, it follows from Assumption 2 that:

(57) $$\begin{array}{c} ∥\delta_{ij}\left(x_{j}\left(t\right)\right)∥\leq \sum_{i∼j}\alpha_{ij}\left[∥{\tilde{x}}_{j}\left(t\right)∥+∥{\hat{e}}_{j}\left(t\right)∥+x_{rj}^{*}\right] \end{array}$$

Furthermore, using Equation (57) in the last term of Equation (56) results in:

(58) $$\begin{array}{cc} ∥2{\tilde{x}}_{i}\left(t\right)P_{i}B_{i}\delta_{ij}\left(x_{j}\left(t\right)\right)∥ & \leq 2\lambda_{\text{max}}\left(P_{i}\right)∥{\tilde{x}}_{i}\left(t\right)∥∥B_{i}{∥}_{F}∥\delta_{ij}\left(x_{j}\left(t\right)\right)∥\\ & \leq 2\lambda_{\text{max}}\left(P_{i}\right)∥{\tilde{x}}_{i}\left(t\right)∥∥B_{i}{∥}_{F}\sum_{i∼j}\alpha_{ij}\left[∥{\tilde{x}}_{j}\left(t\right)∥+∥{\hat{e}}_{j}\left(t\right)∥+x_{rj}^{*}\right]\\ & \leq \lambda_{\text{max}}\left(P_{i}\right){∥B_{i}∥}_{F}\sum_{i∼j}\alpha_{ij}\left[2∥{\tilde{x}}_{i}\left(t\right)∥∥{\tilde{x}}_{j}\left(t\right)∥+2∥{\tilde{x}}_{i}\left(t\right)∥∥{\hat{e}}_{j}\left(t\right)∥+2∥{\tilde{x}}_{i}\left(t\right)∥x_{rj}^{*}\right]\\ & \leq \lambda_{\text{max}}\left(P_{i}\right){∥B_{i}∥}_{F}\sum_{i∼j}\alpha_{ij}\left[3∥{\tilde{x}}_{i}{\left(t\right)∥}^{2}+∥{\tilde{x}}_{j}{\left(t\right)∥}^{2}+{∥{\hat{e}}_{j}\left(t\right)∥}^{2}+{x_{rj}^{*}}^{2}\right] \end{array}$$

where Young’s inequality [46] is considered in the scalar form of $2xy\leq \nu x^{2}+y^{2}/\nu$, with x,y∈R and ν>0, and applied to terms $∥{\tilde{x}}_{i}\left(t\right)∥∥{\tilde{x}}_{j}\left(t\right)∥$, $∥{\tilde{x}}_{i}\left(t\right)∥∥{\hat{e}}_{j}\left(t\right)∥$ and $∥{\tilde{x}}_{i}\left(t\right)∥x_{rj}^{*}$ with ν=1. Hence, Equation (56) becomes:

(59) $$\begin{array}{cc} & {\dot{V}}_{i}\left({\tilde{x}}_{i}\left(t\right),{\tilde{W}}_{i}\left(t\right),{\hat{e}}_{i}\left(t\right)\right)\\ & \leq \;-\underset{d_{1i}}{\underset{︸}{\left[c_{1i}-3\lambda_{\text{max}}\left(P_{i}\right){∥B_{i}∥}_{F}\sum_{i∼j}\alpha_{ij}\right]}}∥{\tilde{x}}_{i}{\left(t\right)∥}^{2}-c_{2i}∥{\hat{e}}_{i}{\left(t\right)∥}^{2}+c_{3i}∥{\tilde{x}}_{i}\left(t\right)∥+c_{4i}∥{\hat{e}}_{i}\left(t\right)∥\\ & \;+\underset{f_{i}}{\underset{︸}{\lambda_{\text{max}}\left(P_{i}\right){∥B_{i}∥}_{F}}}\sum_{i∼j}\alpha_{ij}∥{\tilde{x}}_{j}{\left(t\right)∥}^{2}+\underset{f_{i}}{\underset{︸}{\lambda_{\text{max}}\left(P_{i}\right){∥B_{i}∥}_{F}}}\sum_{i∼j}\alpha_{ij}{∥{\hat{e}}_{j}\left(t\right)∥}^{2}+\varphi_{i} \end{array}$$

where $\varphi_{i}≜c_{5i}+\lambda_{\text{max}}\left(P_{i}\right){∥B_{i}∥}_{F}\sum_{i∼j}\alpha_{ij}{x_{rj}^{*}}^{2}$. Introducing:

(60) $$\begin{array}{c} V\left(\cdot \right)=\sum_{i=1}^{N}V_{i}\left({\tilde{x}}_{i}\left(t\right),{\tilde{W}}_{i}\left(t\right){\hat{e}}_{i}\left(t\right)\right) \end{array}$$

for the uncertain system S results in:

(61) $$\begin{array}{cc} \dot{V}\left(\cdot \right) & \leq \;\sum_{i=1}^{N}[-d_{1i}∥{\tilde{x}}_{i}{\left(t\right)∥}^{2}-c_{2i}∥{\hat{e}}_{i}{\left(t\right)∥}^{2}+c_{3i}∥{\tilde{x}}_{i}\left(t\right)∥\\ & +c_{4i}∥{\hat{e}}_{i}\left(t\right)∥+F_{i}\sum_{i∼j}\alpha_{ij}∥{\tilde{x}}_{j}{\left(t\right)∥}^{2}+F_{i}\sum_{i∼j}\alpha_{ij}{∥{\hat{e}}_{j}\left(t\right)∥}^{2}+\varphi_{i}]\\ & =\;\sum_{i=1}^{N}[-\underset{D_{1i}}{\underset{︸}{\left(d_{1i}-\sum_{i∼j}F_{j}\alpha_{ji}\right)}}∥{\tilde{x}}_{i}{\left(t\right)∥}^{2}-\underset{D_{2i}}{\underset{︸}{\left(c_{2i}-\sum_{i∼j}F_{j}\alpha_{ji}\right)}}{∥{\hat{e}}_{i}\left(t\right)∥}^{2}\\ & +c_{3i}∥{\tilde{x}}_{i}\left(t\right)∥+c_{4i}∥{\hat{e}}_{i}\left(t\right)∥+\varphi_{i}] \end{array}$$

where $D_{1i}>0$ and $D_{2i}>0$ are defined in Assumption 5. Letting ${\tilde{x}}_{a}\left(t\right)≜{\left[∥{\tilde{x}}_{1}\left(t\right)∥,\cdots ,∥{\tilde{x}}_{N}\left(t\right)∥\right]}^{T}$, ${\hat{e}}_{a}\left(t\right)≜{\left[∥{\hat{e}}_{1}\left(t\right)∥,\cdots ,∥{\hat{e}}_{N}\left(t\right)∥\right]}^{T}$, $D_{1}≜\text{diag}\left(\left[D_{11},\cdots ,D_{1N}\right]\right)$, $D_{2}≜\text{diag}\left(\left[D_{21},\cdots ,D_{2N}\right]\right)$, $C_{3}≜\text{diag}\left(\left[c_{31},\cdots ,c_{3N}\right]\right)$, $C_{4}≜\text{diag}\left(\left[c_{41},\cdots ,c_{4N}\right]\right)$ and $\varphi_{a}≜\sum_{i=1}^{N}\varphi_{i}$, then Equation (61) can equivalently be written as:

(62) $$\begin{array}{cc} \dot{V}\left(\cdot \right) & \leq -{\tilde{x}}_{a}^{T}\left(t\right)D_{1}{\tilde{x}}_{a}\left(t\right)-{\hat{e}}_{a}^{T}\left(t\right)D_{2}{\hat{e}}_{a}\left(t\right)+C_{3}{\tilde{x}}_{a}\left(t\right)+C_{4}{\hat{e}}_{a}\left(t\right)+\varphi_{a}\\ & \leq -\lambda_{\text{min}}\left(D_{1}\right)∥{\tilde{x}}_{a}\left(t\right){∥}^{2}-\lambda_{\text{min}}\left(D_{2}\right)∥{\hat{e}}_{a}\left(t\right){∥}^{2}+\lambda_{\text{max}}\left(C_{3}\right)∥{\tilde{x}}_{a}\left(t\right)∥+\lambda_{\text{max}}\left(C_{4}\right)∥{\hat{e}}_{a}\left(t\right)∥+\varphi_{a} \end{array}$$

Either $∥{\tilde{x}}_{a}\left(t\right)∥>\psi_{1}$ or $∥{\hat{e}}_{a}\left(t\right)∥>\psi_{2}$ renders $\dot{V}\left(\cdot \right)<0$, where $\psi_{1}≜\frac{\frac{\lambda_{\text{max}}\left(C_{3}\right)}{2\sqrt{\lambda_{\text{min}}\left(D_{1}\right)}}+\sqrt{\frac{\lambda_{\text{max}}^{2}\left(C_{3}\right)}{4\lambda_{\text{min}}\left(D_{1}\right)}+\frac{\lambda_{\text{max}}^{2}\left(C_{4}\right)}{4\lambda_{\text{min}}\left(D_{2}\right)}+\varphi_{a}}}{\sqrt{\lambda_{\text{min}}\left(D_{1}\right)}}$ and $\psi_{2}≜\frac{\frac{\lambda_{\text{max}}\left(C_{4}\right)}{2\sqrt{\lambda_{\text{min}}\left(D_{2}\right)}}+\sqrt{\frac{\lambda_{\text{max}}^{2}\left(C_{3}\right)}{4\lambda_{\text{min}}\left(D_{1}\right)}+\frac{\lambda_{\text{max}}^{2}\left(C_{4}\right)}{4\lambda_{\text{min}}\left(D_{2}\right)}+\varphi_{a}}}{\sqrt{\lambda_{\text{min}}\left(D_{2}\right)}}$, and hence, ${\tilde{x}}_{i}\left(t\right)$, ${\hat{e}}_{i}\left(t\right)$ and ${\tilde{W}}_{i}\left(t\right)$ are uniformly ultimate bounded for all i=1,2,...,N. ☐

**Corollary 4.**  *Under the conditions of Corollary 3, we can show that $e_{i}\left(t\right)$ is bounded for all i=1,2,...,N.*

**Proof.**  It readily follows from:

(63) $$\begin{array}{cc} ∥e_{i}\left(t\right)∥ & =∥x_{i}\left(t\right)-\hat{x}\left(t\right)+\hat{x}\left(t\right)-x_{r}\left(t\right)∥\\ & \leq ∥x_{i}\left(t\right)-\hat{x}\left(t\right)∥+∥\hat{x}\left(t\right)-x_{r}\left(t\right)∥\\ & \leq ∥{\tilde{x}}_{i}\left(t\right)∥+∥{\hat{e}}_{i}\left(t\right)∥ \end{array}$$

and Corollary 3 that $e_{i}\left(t\right)$ is bounded for all i=1,2,...,N. ☐

**Remark 2.**  *In order to obtain the closed-loop system error ultimate bound value for Equation (63) and the no Zeno behavior characterization, we can follow the same steps highlighted in Corollaries 1 and 2, respectively.*

## 3. Event-Triggered Distributed Adaptive Control

We now introduce an event-triggered distributed adaptive control architecture in this section, where it is assumed that physically-interconnected modules can locally communicate with each other for exchanging their state information. For organizational purposes, this section is broken up into two subsections. Specifically, we first briefly overview a standard distributed adaptive control architecture without event-triggering and then present the proposed event-triggered decentralized adaptive control approach, which includes rigorous stability and performance analyses with no Zeno behavior and generalizations to the state emulator case for suppressing the effect of possible high-frequency oscillations in the controller response. As shown, the benefit of using the proposed distributed adaptive control architecture versus the decentralized architecture of the previous section is that there is no need for any structural assumptions; that is, Assumptions 4 and 5, in the distributed case to guarantee overall system stability (for applications where modules are allowed to locally communicate with each other).

### 3.1. Overview of a Standard Distributed Adaptive Control Architecture without Event-Triggering

The standard distributed adaptive control architecture overviewed in this section builds on the problem formulation stated in Section 2.1 with an important difference that the physically-interconnected modules can locally communicate with each other for exchanging their state information, as discussed above. For this purpose, we first replace Assumption 2 of Section 2.1 with the following assumption.

**Assumption 6.**  *The function $\delta_{ij}\left(x_{j}\left(t\right)\right)$ in Equation (2) satisfies:*

(64) $$\begin{array}{c} \delta_{ij}\left(x_{j}\left(t\right)\right)=Q_{ij}^{T}\phi_{ij}\left(x_{j}\left(t\right)\right) \end{array}$$

*where $Q_{ij}\in R^{g_{i}\times m_{i}}$ is an unknown weight matrix and $\phi_{ij}:R^{n_{j}}\to R^{g_{i}}$ is a known Lipschitz continuous basis function vector satisfying:*

(65) $$\begin{array}{c} ∥\phi_{ij}\left(x_{1j}\right)-\phi_{ij}\left(x_{2j}\right)∥\leq L_{\phi ij}∥x_{1j}-x_{2j}∥ \end{array}$$

*with $L_{\phi ij}\in R_{+}$.*

**Remark 3.**  *We can equivalently represent Equation (64) as:*

(66) $$\begin{array}{c} \sum_{i∼j}Q_{ij}^{T}\phi_{ij}\left(x_{j}\left(t\right)\right)≜G_{ij}^{T}F_{ij}\left(x_{j}\left(t\right)\right) \end{array}$$

*where $G_{ij}\in R^{\left(g_{i}\cdot d_{i}\right)\times m_{i}}$ is the matrix combination for the ideal weight matrices of the connected graph, $F_{ij}\left(x_{j}\left(t\right)\right):R^{n_{j}}\to R^{\left(g_{i}\cdot d_{i}\right)}$ is the vector combination for basis function vectors of the connected graph and $d_{i}$ is the degree of the i-th agent.* *The right hand side of Equation (66) can be given as:*

(67) $$\begin{array}{c} G_{ij}^{T}F_{ij}\left(x_{j}\left(t\right)\right)=G_{i}^{T}\text{diag}\left(A_{i}\right)F_{i} \end{array}$$

*where $G_{i}\in R^{\left(g_{i}\cdot N\right)\times m_{i}}$ is the matrix combination for all modules’ ideal weight matrices of the system toward $S_{i}$, $F_{i}\left(x_{j}\left(t\right)\right):R^{n_{j}}\to R^{\left(g_{i}\cdot N\right)}$ is the vector combination for all basis function vectors of the system toward $S_{i}$ and $A_{i}$ is the i-th row of the adjacency matrix A.*

Next, using Assumptions 1, 3 and 6, Equation (2) can be equivalently written as:

(68) $$\begin{array}{c} {\dot{x}}_{i}\left(t\right)=A_{ri}x_{i}\left(t\right)+B_{ri}c_{i}\left(t\right)+B_{i}\Lambda_{i}\left[u_{i}\left(t\right)+W_{i}^{T}\sigma_{i}\left(x_{i}\left(t\right),c_{i}\left(t\right),x_{j}\left(t\right)\right)\right] \end{array}$$

where $W_{i}≜{\left[\Lambda_{i}^{-1}W_{oi}^{T}\;,\;\Lambda_{i}^{-1}K_{1i}^{T}\;,\;\Lambda_{i}^{-1}K_{2i}^{T}\;,\;\Lambda_{i}^{-1}G_{ij}^{T}\right]}^{T}\in R^{\left(g_{i}+n_{i}+m_{i}+\left(g_{i}\cdot d_{i}\right)\right)\times m_{i}}$ is an unknown weight matrix and $\sigma_{i}\left(x_{i}\left(t\right),c_{i}\left(t\right),x_{j}\left(t\right)\right)≜{\left[\beta_{i}^{T}\left(x_{i}\left(t\right)\right)\;,\;x_{i}^{T}\left(t\right)\;,\;c_{i}^{T}\left(t\right)\;,\;F_{ij}^{T}\left(x_{j}\left(t\right)\right)\right]}^{T}\in R^{g_{i}+n_{i}+m_{i}+\left(g_{i}\cdot d_{i}\right)}$. Motivated from the structure of the uncertain terms appearing in Equation (68), let the distributed adaptive feedback controller of $S_{i},\;i\in V_{G}$, be given by:

(69) $$\begin{array}{c} C_{i}:\;u_{i}\left(t\right)=-{\hat{W}}_{i}{\left(t\right)}^{T}\sigma_{i}\left(x_{i}\left(t\right),c_{i}\left(t\right),x_{j}\left(t\right)\right) \end{array}$$

where ${\hat{W}}_{i}\left(t\right)$ is an estimate of $W_{i}$ satisfying the update law:

(70) $$\begin{array}{c} {\dot{\hat{W}}}_{i}\left(t\right)=\gamma_{i}{\mathrm{Proj}}_{m}[{\hat{W}}_{i}\left(t\right)\;,\;\sigma_{i}\left(x_{i}\left(t\right),c_{i}\left(t\right),x_{j}\left(t\right)\right)e_{i}^{T}\left(t\right)P_{i}B_{i}],\;{\hat{W}}_{i}\left(0\right)={\hat{W}}_{i0} \end{array}$$

where $P_{i}\in R_{+}^{n_{i}\times n_{i}}\cap S^{n_{i}\times n_{i}}$ is a solution of the Lyapunov Equation (10). Now, from Equations (6) and (68), the module-level closed-loop error dynamics can be given by:

(71) $$\begin{array}{c} {\dot{e}}_{i}\left(t\right)=A_{ri}e_{i}\left(t\right)-B_{i}\Lambda_{i}{\tilde{W}}_{i}^{T}\left(t\right)\sigma_{i}\left(x_{i}\left(t\right),c_{i}\left(t\right),x_{j}\left(t\right)\right),\;e_{i}\left(t\right)=e_{i0} \end{array}$$

### 3.2. Proposed Event-Triggered Distributed Adaptive Control Architecture

We now present the proposed event-triggered distributed adaptive control architecture for modular systems, where each uncertain module can exchange its state information with its interconnected neighboring modules.

Consider the uncertain dynamical module *i* given by:

(72) $$\begin{array}{c} S_{i}:\;{\dot{x}}_{i}\left(t\right)=A_{i}x_{i}\left(t\right)+B_{i}\left[\Lambda_{i}u_{si}\left(t\right)+\Delta_{i}\left(x_{i}\left(t\right)\right)+\delta_{ij}\left(x_{sj}\left(t\right)\right)\right],\;x_{i}\left(0\right)=x_{i0} \end{array}$$

where $∥\delta_{ij}\left(x_{sj}\left(t\right)\right)∥\leq \sum_{i∼j}Q_{ij}^{T}\phi_{ij}\left(x_{sj}\left(t\right)\right)$ and $x_{sj}\left(t\right)\in R^{n_{j}}$. Using Assumptions 1, 3 and 6, Equation (72) can be equivalently written as:

(73) $$\begin{array}{cc} {\dot{x}}_{i}\left(t\right)= & A_{ri}x_{i}\left(t\right)+B_{ri}c_{i}\left(t\right)+B_{i}\Lambda_{i}\left[u_{si}\left(t\right)+W_{i}^{T}\sigma_{i}\left(x_{i}\left(t\right),x_{si}\left(t\right),c_{i}\left(t\right),x_{sj}\left(t\right)\right)\right]\\ & +B_{i}\Lambda_{i}\left(u_{si}\left(t\right)-u_{i}\left(t\right)\right)+B_{i}K_{1i}\left(x_{si}\left(t\right)-x_{i}\left(t\right)\right) \end{array}$$

where $\sigma_{i}\left(x_{i}\left(t\right),x_{si}\left(t\right),c_{i}\left(t\right),x_{sj}\left(t\right)\right)≜{\left[\beta_{i}^{T}\left(x_{i}\left(t\right)\right)\;,\;x_{si}^{T}\left(t\right)\;,\;c_{i}^{T}\left(t\right)\;,\;F_{ij}^{T}\left(x_{sj}\left(t\right)\right)\right]}^{T}\in R^{g_{i}+n_{i}+m_{i}+\left(g_{i}\cdot d_{i}\right)}$, and the distributed adaptive feedback control is given by:

(74) $$\begin{array}{c} C_{i}:\;u_{i}\left(t\right)=-{\hat{W}}_{i}{\left(t\right)}^{T}\sigma_{i}\left(x_{si}\left(t\right),c_{i}\left(t\right),x_{sj}\left(t\right)\right) \end{array}$$

where $\sigma_{i}\left(x_{si}\left(t\right),c_{i}\left(t\right),x_{sj}\left(t\right)\right)≜{\left[\beta_{i}^{T}\left(x_{si}\left(t\right)\right)\;,\;x_{si}^{T}\left(t\right)\;,\;c_{i}^{T}\left(t\right)\;,\;F_{ij}^{T}\left(x_{sj}\left(t\right)\right)\right]}^{T}\in R^{g_{i}+n_{i}+m_{i}+g_{i}\cdot d_{i}}$, and ${\hat{W}}_{i}\left(t\right)$ satisfies the weight update law:

(75) $$\begin{array}{c} {\dot{\hat{W}}}_{i}\left(t\right)=\gamma_{i}{\mathrm{Proj}}_{m}[{\hat{W}}_{i}\left(t\right)\;,\;\sigma_{i}\left(x_{si}\left(t\right),c_{i}\left(t\right),x_{sj}\left(t\right)\right)e_{si}^{T}\left(t\right)P_{i}B_{i}],\;{\hat{W}}_{i}\left(0\right)={\hat{W}}_{i0} \end{array}$$

Now, using Equation (74) in Equation (73) yields:

(76) $$\begin{array}{cc} {\dot{x}}_{i}\left(t\right)= & A_{ri}x_{i}\left(t\right)+B_{ri}c_{i}\left(t\right)-B_{i}\Lambda_{i}{\tilde{W}}_{i}^{T}\left(t\right)\sigma_{i}\left(x_{si}\left(t\right),c_{i}\left(t\right),x_{sj}\left(t\right)\right)-B_{i}\Lambda_{i}g_{i}\left(\cdot \right)\\ & +B_{i}\Lambda_{i}\left(u_{si}\left(t\right)-u_{i}\left(t\right)\right)+B_{i}K_{1i}\left(x_{si}\left(t\right)-x_{i}\left(t\right)\right) \end{array}$$

where $g_{i}\left(\cdot \right)≜W_{i}^{T}\left[\sigma_{i}\left(x_{si}\left(t\right),c_{i}\left(t\right),x_{sj}\left(t\right)\right)-\sigma_{i}\left(x_{i}\left(t\right),x_{si}\left(t\right),c_{i}\left(t\right),x_{sj}\left(t\right)\right)\right]$, and using Equations (76) and (6), we can write the module error dynamics as:

(77) $$\begin{array}{cc} {\dot{e}}_{i}\left(t\right)= & A_{ri}e_{i}\left(t\right)-B_{i}\Lambda_{i}{\tilde{W}}_{i}^{T}\left(t\right)\sigma_{i}\left(x_{si}\left(t\right),c_{i}\left(t\right),x_{sj}\left(t\right)\right)-B_{i}\Lambda_{i}g_{i}\left(\cdot \right)+B_{i}\Lambda_{i}\left(u_{si}\left(t\right)-u_{i}\left(t\right)\right)\\ & +B_{i}K_{1i}\left(x_{si}\left(t\right)-x_{i}\left(t\right)\right) \end{array}$$

For organizational purposes, we now divide this section into four sections. Specifically, we analyze the uniform ultimate boundedness of the resulting closed-loop dynamical system in Section 3.2.1, compute the ultimate bound in Section 3.2.2, show that the proposed architecture does not yield to a Zeno behavior in Section 3.2.3 and generalize the distributed event-triggered adaptive control algorithm using the state emulator-based framework in Section 3.2.4.

#### 3.2.1. Stability Analysis and Uniform Ultimate Boundedness

**Theorem** **2.**  *Consider the uncertain dynamical system S consisting of N interconnected modules $S_{i}$ described by Equation (72) subject to Assumptions 1, 3 and 6. Consider, in addition, the reference model given by Equation (6) and the module feedback control law given by Equations (74) and (75). Moreover, let the data transmission from the uncertain dynamical module to the local controller occur when ${\bar{E}}_{1i}$ is true and the data transmission from the controller to the uncertain dynamical system occur when ${\bar{E}}_{2i}\;\vee \;E_{3i}$ is true. Then, the closed-loop solution $\left(e_{i}\left(t\right),{\tilde{W}}_{i}\left(t\right)\right)$ is uniformly ultimately bounded for all i=1,2,...,N.*

**Proof.**  Since the data transmission from the uncertain dynamical module to the local controller and from the local controller to the uncertain dynamical module occur when ${\bar{E}}_{1i}$ and ${\bar{E}}_{2i}\;\vee \;E_{3i}$ are true, respectively, note that $∥x_{si}\left(t\right)-x_{i}\left(t\right)∥\leq \epsilon_{yi}$ and $∥u_{si}\left(t\right)-u_{i}\left(t\right)∥\leq \epsilon_{ui}$ hold. Consider the Lyapunov-like function given by:

(78) $$\begin{array}{c} V_{i}\left(e_{i},{\tilde{W}}_{i}\right)=e_{i}^{T}P_{i}e_{i}+\gamma_{i}^{-1}\text{tr}\left({\left({\tilde{W}}_{i}\Lambda_{i}^{\frac{1}{2}}\right)}^{T}\left(\tilde{W_{i}}\Lambda_{i}^{\frac{1}{2}}\right)\right) \end{array}$$

Note that $V_{i}\left(0,0\right)=0$ and $V_{i}\left(e_{i},{\tilde{W}}_{i}\right)>0$ for all $\left(e_{i},{\tilde{W}}_{i}\right)\neq \left(0,0\right)$. The time derivative of Equation (78) is given by:

(79) $$\begin{array}{c} {\dot{V}}_{i}\left(e_{i}\left(t\right),{\tilde{W}}_{i}\left(t\right)\right)\\ =\;\;2e_{i}^{T}\left(t\right)P{\dot{e}}_{i}\left(t\right)+\gamma_{i}^{-1}2\text{tr}\left({\tilde{W}}_{i}^{T}\left(t\right){\dot{\tilde{W}}}_{i}\left(t\right)\Lambda_{i}\right)\\ \leq \;\;2e_{i}^{T}\left(t\right)P_{i}(A_{ri}e_{i}\left(t\right)-B_{i}\Lambda_{i}{\tilde{W}}_{i}^{T}\left(t\right)\sigma_{i}\left(x_{si}\left(t\right),c_{i}\left(t\right),x_{sj}\left(t\right)\right)-B_{i}\Lambda_{i}g_{i}\left(\cdot \right)+B_{i}\Lambda_{i}\left(u_{si}\left(t\right)-u_{i}\left(t\right)\right)\\ +B_{i}K_{1i}\left(x_{si}\left(t\right)-x_{i}\left(t\right)\right))+2\text{tr}\left({\tilde{W}}_{i}^{T}\left(t\right)\Lambda_{i}\sigma_{i}\left(x_{si}\left(t\right),c_{i}\left(t\right),x_{sj}\left(t\right)\right)e_{si}^{T}\left(t\right)P_{i}B_{i}\right)\\ \leq -e_{i}^{T}\left(t\right)R_{i}e_{i}\left(t\right)-2e_{i}^{T}\left(t\right)P_{i}B_{i}\Lambda_{i}g_{i}\left(\cdot \right)+2e_{i}^{T}\left(t\right)P_{i}B_{i}\Lambda_{i}\left(u_{si}\left(t\right)-u_{i}\left(t\right)\right)+2e_{i}^{T}\left(t\right)P_{i}B_{i}K_{1i}\left(x_{si}\left(t\right)-x_{i}\left(t\right)\right)\\ +2\text{tr}\left({\tilde{W}}_{i}^{T}\left(t\right)\Lambda_{i}\sigma_{i}\left(x_{si}\left(t\right),c_{i}\left(t\right),x_{sj}\left(t\right)\right){\left(x_{si}\left(t\right)-x_{i}\left(t\right)\right)}^{T}P_{i}B_{i}\right)\\ \leq -\lambda_{\text{min}}\left(R_{i}\right)∥e_{i}\left(t\right){∥}^{2}+2∥e_{i}\left(t\right)∥\lambda_{\text{max}}\left(P_{i}\right)∥B_{i}{∥}_{F}∥\Lambda_{i}{∥}_{F}∥g_{i}\left(\cdot \right)∥+2∥e_{i}\left(t\right)∥\lambda_{\text{max}}\left(P_{i}\right){∥B_{i}∥}_{F}\\ \cdot ∥\Lambda_{i}{∥}_{F}\epsilon_{ui}+2∥e_{i}\left(t\right)∥\lambda_{\text{max}}\left(P_{i}\right)∥B_{i}{∥}_{F}∥K_{1i}{∥}_{F}\epsilon_{xi}+2∥{\tilde{W}}_{i}\left(t\right){∥}_{F}∥\Lambda_{i}{∥}_{F}∥\sigma_{i}\left(x_{si}\left(t\right),c_{i}\left(t\right),x_{sj}\left(t\right)\right)∥\\ \cdot \epsilon_{xi}\lambda_{\text{max}}\left(P_{i}\right){∥B_{i}∥}_{F} \end{array}$$

where the same upper bound $∥g_{i}\left(\cdot \right)∥$ has the same result of Equation (25). In addition, one can compute an upper bound for $∥\sigma_{i}\left(x_{si}\left(t\right),c_{i}\left(t\right),x_{sj}\left(t\right)\right)∥$ in Equation (79) as:

(80) $$\begin{array}{cc} ∥\sigma_{i}\left(x_{si}\left(t\right),c_{i}\left(t\right),x_{sj}\left(t\right)\right)∥ & \leq ∥\beta_{i}\left(x_{si}\left(t\right)\right)∥+∥x_{si}\left(t\right)∥+∥c_{i}\left(t\right)∥+∥F_{ij}\left(x_{sj}\left(t\right)\right)∥\\ & \leq L_{\beta i}∥x_{si}\left(t\right)∥+∥x_{si}\left(t\right)∥+∥c_{i}\left(t\right)∥+\sum_{i∼j}∥\phi_{ij}\left(x_{j}\left(t\right)\right)∥\\ & =\left(L_{\beta i}+1\right)\epsilon_{xi}+\left(L_{\beta i}+1\right)∥e_{i}\left(t\right)∥+\left(L_{\beta i}+1\right)x_{ri}^{*}+∥c_{i}\left(t\right)∥\\ & \;+\sum_{i∼j}L_{\phi ij}\left(\epsilon_{xj}+∥e_{j}\left(t\right)∥+x_{rj}^{*}\right) \end{array}$$

where $∥x_{ri}\left(t\right)∥\leq x_{ri}^{*}$ and $∥x_{rj}\left(t\right)∥\leq x_{rj}^{*}$. Then, using the bounds given by Equations (25) and (80) in Equation (79) yields:

(81) $$\begin{array}{c} {\dot{V}}_{i}\left(e_{i}\left(t\right),{\tilde{W}}_{i}\left(t\right)\right)\\ \leq \;\;-\lambda_{\text{min}}\left(R_{i}\right)∥e_{i}\left(t\right){∥}^{2}+2∥e_{i}\left(t\right)∥\lambda_{\text{max}}\left(P_{i}\right)∥B_{i}{∥}_{F}∥\Lambda_{i}{∥}_{F}K_{gi}\epsilon_{xi}+2∥e_{i}\left(t\right)∥\lambda_{\text{max}}\left(P_{i}\right)∥B_{i}{∥}_{F}{∥\Lambda_{i}∥}_{F}\epsilon_{ui}\\ +2∥e_{i}\left(t\right)∥\lambda_{\text{max}}\left(P_{i}\right)∥B_{i}{∥}_{F}∥K_{1i}{∥}_{F}\epsilon_{xi}+2∥{\tilde{W}}_{i}\left(t\right){∥}_{F}∥\Lambda_{i}{∥}_{F}(\left(L_{\beta i}+1\right)\epsilon_{xi}+\left(L_{\beta i}+1\right)∥e_{i}\left(t\right)∥\\ +\left(L_{\beta i}+1\right)x_{ri}^{*}+∥c_{i}\left(t\right)∥+\sum_{i∼j}L_{\phi ij}\left(\epsilon_{xj}+∥e_{j}\left(t\right)∥+x_{rj}^{*}\right))\epsilon_{xi}\lambda_{\text{max}}\left(P_{i}\right){∥B_{i}∥}_{F}\\ \leq \;\;-\lambda_{\text{min}}\left(R_{i}\right)∥e_{i}\left(t\right){∥}^{2}+(2\lambda_{\text{max}}\left(P_{i}\right)∥B_{i}{∥}_{F}∥\Lambda_{i}{∥}_{F}K_{gi}\epsilon_{xi}+2\lambda_{\text{max}}\left(P_{i}\right)∥B_{i}{∥}_{F}{∥\Lambda_{i}∥}_{F}\epsilon_{ui}\\ +2\lambda_{\text{max}}\left(P_{i}\right)∥B_{i}{∥}_{F}∥K_{1i}{∥}_{F}\epsilon_{xi}+2{\tilde{w}}_{i}^{*}∥\Lambda_{i}{∥}_{F}\left(L_{\beta i}+1\right)\epsilon_{xi}\lambda_{\text{max}}\left(P_{i}\right)∥B_{i}{∥}_{F})∥e_{i}\left(t\right)∥\\ +2{\tilde{w}}_{i}^{*}∥\Lambda_{i}{∥}_{F}\left(\left(L_{\beta i}+1\right)\epsilon_{xi}+\left(L_{\beta i}+1\right)x_{ri}^{*}+∥c_{i}\left(t\right)∥+\sum_{i∼j}L_{\phi ij}\left(\epsilon_{xj}+x_{rj}^{*}\right)\right)\epsilon_{xi}\lambda_{\text{max}}\left(P_{i}\right){∥B_{i}∥}_{F}\\ +2{\tilde{w}}_{i}^{*}∥\Lambda_{i}{∥}_{F}\epsilon_{xi}\lambda_{\text{max}}\left(P_{i}\right)∥B_{i}{∥}_{F}\sum_{i∼j}L_{\phi ij}∥e_{j}\left(t\right)∥\\ \leq \;\;-d_{1i}∥e_{i}{\left(t\right)∥}^{2}+d_{2i}∥e_{i}\left(t\right)∥+d_{3i}+F_{i}\sum_{i∼j}L_{\phi ij}∥e_{j}\left(t\right)∥ \end{array}$$

where $d_{1i}≜\lambda_{\text{min}}\left(R_{i}\right)$, $d_{2i}≜2\lambda_{\text{max}}\left(P_{i}\right)∥B_{i}{∥}_{F}∥\Lambda_{i}{∥}_{F}K_{gi}\epsilon_{xi}+2\lambda_{\text{max}}\left(P_{i}\right)∥B_{i}{∥}_{F}∥\Lambda_{i}{∥}_{F}\epsilon_{ui}+2\lambda_{\text{max}}\left(P_{i}\right)∥B_{i}{∥}_{F}{∥K_{1i}∥}_{F}\epsilon_{xi}+2{\tilde{w}}_{i}^{*}∥\Lambda_{i}{∥}_{F}\left(L_{\beta i}+1\right)\epsilon_{xi}\lambda_{\text{max}}\left(P_{i}\right){∥B_{i}∥}_{F}$, $d_{3i}≜2{\tilde{w}}_{i}^{*}∥\Lambda_{i}{∥}_{F}\left(\left(L_{\beta i}+1\right)\epsilon_{xi}+\left(L_{\beta i}+1\right)x_{ri}^{*}+∥c_{i}\left(t\right)∥+\sum_{i∼j}L_{\phi ij}\left(\epsilon_{xj}+x_{rj}^{*}\right)\right)\epsilon_{xi}\lambda_{\text{max}}\left(P_{i}\right){∥B_{i}∥}_{F}$ and $f_{i}≜2{\tilde{w}}_{i}^{*}∥\Lambda_{i}{∥}_{F}\epsilon_{xi}\lambda_{\text{max}}\left(P_{i}\right){∥B_{i}∥}_{F}$.

Introducing:

(82) $$\begin{array}{c} V\left(\cdot \right)=\sum_{i=1}^{N}V_{i}\left(e_{i}\left(t\right),{\tilde{W}}_{i}\left(t\right)\right) \end{array}$$

for the uncertain system S results in:

(83) $$\begin{array}{cc} \dot{V}\left(\cdot \right) & \leq \;\sum_{i=1}^{N}\left[-d_{1i}∥e_{i}{\left(t\right)∥}^{2}+d_{2i}∥e_{i}\left(t\right)∥+f_{i}\sum_{i∼j}L_{\phi ij}∥e_{j}\left(t\right)∥+d_{3i}\right]\\ & =\;\sum_{i=1}^{N}[-d_{1i}∥e_{i}{\left(t\right)∥}^{2}+\underset{D_{2i}}{\underset{︸}{\left(d_{2i}+\sum_{i∼j}f_{j}L_{\phi ji}\right)}}∥e_{i}\left(t\right)∥+d_{3i}] \end{array}$$

where $D_{1i}>0$. Letting $e_{a}\left(t\right)≜{\left[∥e_{1}\left(t\right)∥,\cdots ,∥e_{N}\left(t\right)∥\right]}^{T}$, $D_{1}≜\text{diag}\left(\left[d_{11},\cdots ,d_{1N}\right]\right)$, $D_{2}≜\text{diag}\left(\left[D_{21},\cdots ,D_{2N}\right]\right)$, and $D_{3}≜\sum_{i=1}^{N}d_{3i}$, then Equation (32) can equivalently be written as:

(84) $$\begin{array}{cc} \dot{V}\left(\cdot \right) & \leq -e_{a}^{T}\left(t\right)D_{1}e_{a}\left(t\right)+D_{2}e_{a}\left(t\right)+D_{3}\\ & \leq -\lambda_{\text{min}}\left(D_{1}\right)∥e_{a}\left(t\right){∥}^{2}+\lambda_{\text{max}}\left(D_{2}\right)∥e_{a}\left(t\right)∥+D_{3} \end{array}$$

When $∥e_{a}\left(t\right)∥>\psi$, this renders $\dot{V}\left(\cdot \right)<0$, where $\psi ≜\frac{\frac{\lambda_{\text{max}}\left(D_{2}\right)}{2\sqrt{\lambda_{\text{min}}\left(D_{1}\right)}}+\sqrt{\frac{\lambda_{\text{max}}^{2}\left(D_{2}\right)}{4\lambda_{\text{min}}\left(D_{1}\right)}+D_{3}}}{\sqrt{\lambda_{\text{min}}\left(D_{1}\right)}}$, and hence, $e_{i}\left(t\right)$ and ${\tilde{W}}_{i}\left(t\right)$ are uniformly ultimate bounded for all i=1,2,...,N. ☐

#### 3.2.2. Computation of the Ultimate Bound for System Performance Assessment

For revealing the effect of user-defined thresholds and the event-triggered output feedback adaptive controller design parameters to the system performance, the next corollary presents a computation of the ultimate bound.

**Corollary 5.**  *Consider the uncertain dynamical system S consisting of N interconnected modules $S_{i}$ described by Equation (72) subject to Assumptions 1, 3 and 6. Consider, in addition, the reference model given by Equation (6) and the module feedback control law given by Equations (74) and (75). Moreover, let the data transmission from the uncertain dynamical module to the local controller occur when ${\bar{E}}_{1i}$ is true and the data transmission from the controller to the uncertain dynamical system occur when ${\bar{E}}_{2i}\vee E_{3i}$ is true. Then, the ultimate bound of the system error between the uncertain dynamical system and the reference model is given by:*

(85) $$\begin{array}{c} \left|\right|e_{a}\left(t\right)\left|\right|\;\;\leq \;\;\tilde{\Phi }\lambda_{\min}^{-\frac{1}{2}}\left(P_{\min}\right),\;t\geq T \end{array}$$

*where*

(86) $$\begin{array}{c} \tilde{\Phi }≜[\lambda_{\max}\left(P_{\max}\right)\psi^{2}+\lambda_{\max}\left(\gamma_{a}\right)\lambda_{\max}\left(\Lambda_{a}\right){∥{\tilde{W}}_{a}\left(t\right)∥}^{2}{]}^{\frac{1}{2}}\;\;\;\; \end{array}$$

**Proof.**  The proof is similar to the proof of Corollary 1, and hence, omitted. ☐

#### 3.2.3. Computation of the Event-Triggered Inter-Sample Time Lower Bound

In this subsection, we show that the proposed event-triggered distributed adaptive control architecture does not yield to a Zeno behavior, which implies that it does not require a continuous two-way data exchange and reduces wireless network utilization. For this purpose, we use the same mathematical notations introduced in Section 2.2.2 and make the following assumption.

**Assumption 7.**  *Each module $S_{i}$ holds the received triggered state information $\delta_{ij}\left(x_{sj}\left(t\right)\right)$ from its interconnected neighboring modules $S_{j}$ and sends this information to its local controller $C_{i}$ when the condition $E_{1i}$ in Equation (20) is violated.*

**Corollary 6.**  *Consider the uncertain dynamical system S consisting of N interconnected modules $S_{i}$ described by Equation (72) subject to Assumptions 1, 3, 6 and 7. Consider, in addition, the reference model given by Equation (6) and the module feedback control law given by Equations (74) and (75). Moreover, let the data transmission from the uncertain dynamical module to the local controller occur when ${\bar{E}}_{1i}$ is true and the data transmission from the controller to the uncertain dynamical system occur when ${\bar{E}}_{2i}\vee E_{3i}$ is true. Then, there exist positive scalars $\alpha_{xi}≜\frac{\epsilon_{xi}}{\Phi_{1i}}$ and $\alpha_{ui}≜\frac{\epsilon_{ui}}{\Phi_{2i}}$, such that:*

(87) $$\begin{array}{ccc} s_{k_{i}+1}-s_{k_{i}} & > & \alpha_{xi},\;\forall k_{i}\in N \end{array}$$

(88) $$\begin{array}{ccc} r_{q_{i}+1}^{k_{i}}-r_{q_{i}}^{k} & > & \alpha_{ui},\;\forall q_{i}\in \left\{0,...,m_{k_{i}}\right\},\;\forall k_{i}\in N \end{array}$$

**Proof.**  The proof is similar to the proof of Corollary 2, and hence, omitted. ☐

Corollary 6 also shows that the inter-sample times for the module state vector and distributed feedback control vector are bounded away from zero, and hence, the proposed event-triggered distributed adaptive control approach does not yield to a Zeno behavior.

#### 3.2.4. Generalizations to the Event-Triggered Distributed Adaptive Control with State Emulator

Similar to Section 2.2.4, consider the (modified) reference model, so-called the state emulator, given by Equation (44) and the reference model error dynamics capturing the difference between the ideal reference model Equation (6), and the state emulator-based (modified) reference model Equation (44) is given by Equation (45). In addition, the (state emulator-based) system error dynamics follow from Equations (76) and (44) as:

(89) $$\begin{array}{ccc} {\dot{\tilde{x}}}_{i}\left(t\right) & = & A_{ri}{\tilde{x}}_{i}\left(t\right)-B_{i}\Lambda_{i}{\tilde{W}}_{i}^{T}\left(t\right)\sigma_{i}\left(x_{si}\left(t\right),c_{i}\left(t\right),x_{sj}\left(t\right)\right)-B_{i}\Lambda_{i}g_{i}\left(\cdot \right)\\ & & +B_{i}\Lambda_{i}\left(u_{si}\left(t\right)-u_{i}\left(t\right)\right)+\left(B_{i}K_{1i}-L_{i}\right)\left(x_{si}\left(t\right)-x_{i}\left(t\right)\right)-L_{i}{\tilde{x}}_{i}\left(t\right),\;{\tilde{x}}_{i}\left(0\right)={\tilde{x}}_{i0}\;\; \end{array}$$

where the adaptive controller Equation (74) is used and the weight update law is given by:

(90) $$\begin{array}{c} {\dot{\hat{W}}}_{i}\left(t\right)=\gamma_{i}{\mathrm{Proj}}_{m}[{\hat{W}}_{i}\left(t\right)\;,\;\sigma_{i}\left(x_{si}\left(t\right),c_{i}\left(t\right),x_{sj}\left(t\right)\right){\left(x_{si}\left(t\right)-{\hat{x}}_{i}\left(t\right)\right)}^{T}P_{i}B_{i}],\;{\hat{W}}_{i}\left(0\right)={\hat{W}}_{i0} \end{array}$$

with $P_{i}\in R_{+}^{n_{i}\times n_{i}}\cap S^{n_{i}\times n_{i}}$ being a solution to the Lyapunov Equation (10).

**Corollary 7.**  *Consider the uncertain dynamical system S consisting of N interconnected modules $S_{i}$ described by Equation (72) subject to Assumptions 1, 3 and 6. Consider, in addition, the ideal reference model given by Equation (6), the state emulator given by Equation (44) and the module feedback control law given by Equations (74) and (90). Moreover, let the data transmission from the uncertain dynamical module to the local controller occur when ${\bar{E}}_{1i}$ is true and the data transmission from the controller to the uncertain dynamical system occur when ${\bar{E}}_{2i}\vee E_{3i}$ is true. Then, the closed-loop solution $\left({\tilde{x}}_{i}\left(t\right),{\tilde{W}}_{i}\left(t\right),{\hat{e}}_{i}\left(t\right)\right)$ is uniformly ultimately bounded for all i=1,2,...,N.*

**Proof.**  Consider the Lyapunov-like function given by:

(91) $$\begin{array}{c} V_{i}\left({\tilde{x}}_{i},{\tilde{W}}_{i},{\hat{e}}_{i}\right)={\tilde{x}}_{i}^{T}P_{i}{\tilde{x}}_{i}+\gamma_{i}^{-1}\mathrm{tr}{\left({\tilde{W}}_{i}\Lambda_{i}^{\frac{1}{2}}\right)}^{T}\left({\tilde{W}}_{i}\Lambda_{i}^{\frac{1}{2}}\right)+2l_{i}{∥L_{i}∥}_{F}^{-1}\lambda_{\text{max}}\left(P_{i}\right)\lambda_{\text{max}}\left(R_{i}\right){\hat{e}}_{i}^{T}P_{i}{\hat{e}}_{i} \end{array}$$

Note that $V_{i}\left(0,0,0\right)=0$ and $V_{i}\left({\tilde{x}}_{i},{\tilde{W}}_{i},{\hat{e}}_{i}\right)>0$ for all $\left({\tilde{x}}_{i},{\tilde{W}}_{i},{\hat{e}}_{i}\right)\neq \left(0,0,0\right)$. The time-derivative of Equation (91) is given by:

(92) $$\begin{array}{c} {\dot{V}}_{i}\left({\tilde{x}}_{i}\left(t\right),{\tilde{W}}_{i}\left(t\right),{\hat{e}}_{i}\left(t\right)\right)\\ =\;2{\tilde{x}}_{i}^{T}\left(t\right)P_{i}{\dot{\tilde{x}}}_{i}\left(t\right)+2\gamma_{i}^{-1}\mathrm{tr}{\left({\tilde{W}}_{i}\left(t\right)\Lambda_{i}^{\frac{1}{2}}\right)}^{T}\left({\dot{\tilde{W}}}_{i}\left(t\right)\Lambda_{i}^{\frac{1}{2}}\right)+4l_{i}{∥L_{i}∥}_{F}^{-1}\lambda_{\text{max}}\left(P_{i}\right)\lambda_{\text{min}}\left(R_{i}\right){\hat{e}}_{i}^{T}P_{i}{\dot{\hat{e}}}_{i}\left(t\right)\\ \leq \;2{\tilde{x}}_{i}^{T}\left(t\right)P_{i}[A_{ri}{\tilde{x}}_{i}\left(t\right)-B_{i}\Lambda_{i}{\tilde{W}}_{i}^{T}\left(t\right)\sigma_{i}\left(x_{si}\left(t\right),c_{i}\left(t\right),x_{sj}\left(t\right)\right)-B_{i}\Lambda_{i}g_{i}\left(\cdot \right)+B_{i}\Lambda_{i}\left(u_{si}\left(t\right)-u_{i}\left(t\right)\right)\\ +\left(B_{i}K_{1i}-L_{i}\right)\left(x_{si}\left(t\right)-x_{i}\left(t\right)\right)-L_{i}{\tilde{x}}_{i}\left(t\right)]+2\mathrm{tr}{\tilde{W}}_{i}^{T}\left(t\right)\sigma_{i}\left(x_{si}\left(t\right),c_{i}\left(t\right),x_{sj}\left(t\right)\right){\left(x_{si}\left(t\right)-{\hat{x}}_{i}\left(t\right)\right)}^{T}P_{i}B_{i}\Lambda_{i}\\ +4l_{i}{∥L_{i}∥}_{F}^{-1}\lambda_{\text{max}}\left(P_{i}\right)\lambda_{\text{min}}\left(R_{i}\right){\hat{e}}_{i}^{T}\left(t\right)P_{i}\left[A_{ri}{\hat{e}}_{i}\left(t\right)+L_{i}{\tilde{x}}_{i}\left(t\right))+L_{i}\left(x_{si}\left(t\right)-x_{i}\left(t\right)\right)\right]\\ \leq \;-{\tilde{x}}_{i}^{T}\left(t\right)R_{i}{\tilde{x}}_{i}\left(t\right)-2{\tilde{x}}_{i}^{T}\left(t\right)P_{i}B_{i}\Lambda_{i}g_{i}\left(\cdot \right)+2{\tilde{x}}_{i}^{T}\left(t\right)P_{i}B_{i}\Lambda_{i}\left(u_{si}\left(t\right)-u_{i}\left(t\right)\right)+2{\tilde{x}}_{i}^{T}\left(t\right)P_{i}\left(B_{i}K_{1i}-L_{i}\right)\\ \cdot \left(x_{si}\left(t\right)-x_{i}\left(t\right)\right)-2{\tilde{x}}_{i}^{T}\left(t\right)P_{i}L_{i}{\tilde{x}}_{i}\left(t\right)+2\mathrm{tr}{\tilde{W}}_{i}{\left(t\right)}^{T}\sigma_{i}\left(x_{si}\left(t\right),c_{i}\left(t\right),x_{sj}\left(t\right)\right){\left(x_{si}\left(t\right)-x_{i}\left(t\right)\right)}^{T}P_{i}B_{i}\Lambda_{i}\\ -2l_{i}∥L_{i}{∥}_{F}^{-1}\lambda_{\text{max}}\left(P_{i}\right)\lambda_{\text{min}}\left(R_{i}\right){\hat{e}}_{i}^{T}\left(t\right)R_{i}{\hat{e}}_{i}\left(t\right)+4l_{i}{∥L_{i}∥}_{F}^{-1}\lambda_{\text{max}}\left(P_{i}\right)\lambda_{\text{min}}\left(R_{i}\right){\hat{e}}_{i}^{T}\left(t\right)P_{i}L_{i}\left(x_{si}\left(t\right)-x_{i}\left(t\right)\right)\\ +4l_{i}{∥L_{i}∥}_{F}^{-1}\lambda_{\text{max}}\left(P_{i}\right)\lambda_{\text{min}}\left(R_{i}\right){\hat{e}}_{i}^{T}\left(t\right)P_{i}L_{i}{\tilde{x}}_{i}\left(t\right)\\ \leq \;-\lambda_{\text{min}}\left(R_{i}\right)∥{\tilde{x}}_{i}\left(t\right){∥}^{2}+2\lambda_{\text{max}}\left(P_{i}\right)∥B_{i}{∥}_{F}∥\Lambda_{i}{∥}_{F}∥g_{i}\left(\cdot \right)∥∥{\tilde{x}}_{i}\left(t\right)∥+2∥{\tilde{x}}_{i}\left(t\right)∥\lambda_{\text{max}}\left(P_{i}\right)∥B_{i}{∥}_{F}{∥\Lambda_{i}∥}_{F}\epsilon_{ui}\\ +2∥{\tilde{x}}_{i}\left(t\right)∥\lambda_{\text{max}}\left(P_{i}\right)\left(∥B_{i}K_{1i}{∥}_{F}+{∥L_{i}∥}_{F}\right)\epsilon_{xi}-2\lambda_{\text{max}}\left(P_{i}\right)∥L_{i}∥∥{\tilde{x}}_{i}\left(t\right){∥}^{2}+2{∥{\tilde{W}}_{i}\left(t\right)∥}_{F}\\ \cdot ∥\sigma_{i}\left(x_{si}\left(t\right),c_{i}\left(t\right),x_{sj}\left(t\right)\right)∥\lambda_{\text{max}}\left(P_{i}\right)∥B_{i}{∥}_{F}∥\Lambda_{i}{∥}_{F}\epsilon_{xi}-2l_{i}∥L_{i}{∥}_{F}^{-1}\lambda_{\text{max}}^{-1}\left(P_{i}\right)\lambda_{\text{min}}^{2}\left(R_{i}\right){∥{\hat{e}}_{i}\left(t\right)∥}^{2}\\ +4l_{i}\lambda_{\text{min}}\left(R_{i}\right)\epsilon_{xi}∥{\hat{e}}_{i}\left(t\right)∥+4l_{i}\lambda_{\text{min}}\left(R_{i}\right)∥{\hat{e}}_{i}\left(t\right)∥∥{\tilde{x}}_{i}\left(t\right)∥ \end{array}$$

Now, using Young’s inequality [46] for the last term in Equation (92), with $\mu_{i}\in R_{+}$, yields:

(93) $$\begin{array}{c} {\dot{V}}_{i}\left({\tilde{x}}_{i}\left(t\right),{\tilde{W}}_{i}\left(t\right),{\hat{e}}_{i}\left(t\right)\right)\\ \leq \;-\lambda_{\text{min}}\left(R_{i}\right)∥{\tilde{x}}_{i}\left(t\right){∥}^{2}+2\lambda_{\text{max}}\left(P_{i}\right)∥B_{i}{∥}_{F}∥\Lambda_{i}{∥}_{F}∥g_{i}\left(\cdot \right)∥∥{\tilde{x}}_{i}\left(t\right)∥+2∥{\tilde{x}}_{i}\left(t\right)∥\lambda_{\text{max}}\left(P_{i}\right)∥B_{i}{∥}_{F}{∥\Lambda_{i}∥}_{F}\epsilon_{ui}\\ +2∥{\tilde{x}}_{i}\left(t\right)∥\lambda_{\text{max}}\left(P_{i}\right)\left(∥B_{i}K_{1i}{∥}_{F}+{∥L_{i}∥}_{F}\right)\epsilon_{xi}-2\lambda_{\text{max}}\left(P_{i}\right)∥L_{i}∥∥{\tilde{x}}_{i}\left(t\right){∥}^{2}+2{∥{\tilde{W}}_{i}\left(t\right)∥}_{F}\\ \cdot ∥\sigma_{i}\left(x_{si}\left(t\right),c_{i}\left(t\right),x_{sj}\left(t\right)\right)∥\lambda_{\text{max}}\left(P_{i}\right)∥B_{i}{∥}_{F}∥\Lambda_{i}{∥}_{F}\epsilon_{xi}-2l_{i}∥L_{i}{∥}_{F}^{-1}\lambda_{\text{max}}^{-1}\left(P_{i}\right)\lambda_{\text{min}}^{2}\left(R_{i}\right){∥{\hat{e}}_{i}\left(t\right)∥}^{2}\\ +4l_{i}\lambda_{\text{min}}\left(R_{i}\right)\epsilon_{xi}∥{\hat{e}}_{i}\left(t\right)∥+2l_{i}\mu_{i}\lambda_{\text{min}}\left(R_{i}\right)∥{\hat{e}}_{i}\left(t\right){∥}^{2}+2\frac{l_{i}}{\mu_{i}}\lambda_{\text{min}}\left(R_{i}\right){∥{\tilde{x}}_{i}\left(t\right)∥}^{2} \end{array}$$

Using Equations (25) and (79), Equation (93) can be written by:

(94) $$\begin{array}{c} {\dot{V}}_{i}\left({\tilde{x}}_{i}\left(t\right),{\tilde{W}}_{i}\left(t\right),{\hat{e}}_{i}\left(t\right)\right)\\ \leq \;-\left[\lambda_{\text{min}}\left(R_{i}\right)-2\lambda_{\text{max}}\left(P_{i}\right){∥L_{i}∥}_{F}-2\frac{l_{i}}{\mu_{i}}\lambda_{\text{min}}\left(R_{i}\right)\right]∥{\tilde{x}}_{i}{\left(t\right)∥}^{2}-2[l_{i}{∥L_{i}∥}_{F}^{-1}\lambda_{\text{max}}^{-1}\left(P_{i}\right)\lambda_{\text{min}}^{2}\left(R_{i}\right)\\ -l_{i}\mu_{i}\lambda_{\text{min}}\left(R_{i}\right)]∥{\hat{e}}_{i}\left(t\right){∥}^{2}+[2\lambda_{\text{max}}\left(P_{i}\right)∥B_{i}{∥}_{F}∥\Lambda_{i}{∥}_{F}K_{gi}\epsilon_{xi}+2\lambda_{\text{max}}\left(P_{i}\right)∥B_{i}{∥}_{F}{∥\Lambda_{i}∥}_{F}\epsilon_{ui}\\ +2\lambda_{\text{max}}\left(P_{i}\right)\left(∥B_{i}K_{1i}{∥}_{F}+{∥L_{i}∥}_{F}\right)\epsilon_{xi}]∥{\tilde{x}}_{i}\left(t\right)∥+4l_{i}\lambda_{\text{min}}\left(R_{i}\right)\epsilon_{xi}∥{\hat{e}}_{i}\left(t\right)∥\\ +2{\tilde{w}}_{i}^{*}[\left(L_{\beta i}+1\right)\epsilon_{xi}+\left(L_{\beta i}+1\right)∥{\tilde{x}}_{i}\left(t\right)+{\hat{e}}_{i}\left(t\right)∥+\left(L_{\beta i}+1\right)x_{ri}^{*}+∥c_{i}\left(t\right)∥+\sum_{i∼j}L_{\phi ij}(\epsilon_{xj}\\ +∥{\tilde{x}}_{j}\left(t\right)+{\hat{e}}_{j}\left(t\right)∥+x_{rj}^{*})]\lambda_{\text{max}}\left(P_{i}\right)∥B_{i}{∥}_{F}{∥\Lambda_{i}∥}_{F}\epsilon_{xi}\\ \leq \;-\left[\lambda_{\text{min}}\left(R_{i}\right)-2\lambda_{\text{max}}\left(P_{i}\right){∥L_{i}∥}_{F}-2\frac{l_{i}}{\mu_{i}}\lambda_{\text{min}}\left(R_{i}\right)\right]∥{\tilde{x}}_{i}{\left(t\right)∥}^{2}-2[l_{i}{∥L_{i}∥}_{F}^{-1}\lambda_{\text{max}}^{-1}\left(P_{i}\right)\lambda_{\text{min}}^{2}\left(R_{i}\right)\\ -l_{i}\mu_{i}\lambda_{\text{min}}\left(R_{i}\right)]∥{\hat{e}}_{i}\left(t\right){∥}^{2}+[2\lambda_{\text{max}}\left(P_{i}\right)∥B_{i}{∥}_{F}∥\Lambda_{i}{∥}_{F}K_{gi}\epsilon_{xi}+2\lambda_{\text{max}}\left(P_{i}\right)∥B_{i}{∥}_{F}{∥\Lambda_{i}∥}_{F}\epsilon_{ui}\\ +2\lambda_{\text{max}}\left(P_{i}\right)\left(∥B_{i}K_{1i}{∥}_{F}+{∥L_{i}∥}_{F}\right)\epsilon_{xi}+2{\tilde{w}}_{i}^{*}\lambda_{\text{max}}\left(P_{i}\right)∥B_{i}{∥}_{F}∥\Lambda_{i}{∥}_{F}\epsilon_{xi}]∥{\tilde{x}}_{i}\left(t\right)∥\\ +\left[4l_{i}\lambda_{\text{min}}\left(R_{i}\right)\epsilon_{xi}+2{\tilde{w}}_{i}^{*}\lambda_{\text{max}}\left(P_{i}\right)∥B_{i}{∥}_{F}{∥\Lambda_{i}∥}_{F}\epsilon_{xi}\right]∥{\hat{e}}_{i}\left(t\right)∥\\ +2{\tilde{w}}_{i}^{*}\lambda_{\text{max}}\left(P_{i}\right)∥B_{i}{∥}_{F}{∥\Lambda_{i}∥}_{F}\epsilon_{xi}\left(\left(L_{\beta i}+1\right)\left(\epsilon_{xi}+x_{ri}^{*}\right)+∥c_{i}\left(t\right)∥+\sum_{i∼j}L_{\phi ij}\left(\epsilon_{xj}+x_{rj}^{*}\right)\right)\\ +2{\tilde{w}}_{i}^{*}\lambda_{\text{max}}\left(P_{i}\right)∥B_{i}{∥}_{F}{∥\Lambda_{i}∥}_{F}\epsilon_{xi}\sum_{i∼j}L_{\phi ij}\left(∥{\tilde{x}}_{j}\left(t\right)∥+∥{\hat{e}}_{j}\left(t\right)∥\right) \end{array}$$

then setting $\mu_{i}=l_{i}\lambda_{\text{min}}\left(R_{i}\right)\lambda_{\text{max}}^{-1}\left(P_{i}\right){∥L_{i}∥}_{F}^{-1}$ in Equation (94) yields:

(95) $$\begin{array}{cc} & {\dot{V}}_{i}\left({\tilde{x}}_{i}\left(t\right),{\tilde{W}}_{i}\left(t\right),{\hat{e}}_{i}\left(t\right)\right)\\ & \leq \;-\lambda_{\text{min}}\left(R_{i}\right)∥{\tilde{x}}_{i}\left(t\right){∥}^{2}-2l_{i}∥L_{i}{∥}_{F}^{-1}\lambda_{\text{max}}^{-1}\left(P_{i}\right)\lambda_{\text{min}}\left(R_{i}\right)\left[\lambda_{\text{min}}\left(R_{i}\right)-l_{i}\right]{∥{\hat{e}}_{i}\left(t\right)∥}^{2}\\ & +[2\lambda_{\text{max}}\left(P_{i}\right)∥B_{i}{∥}_{F}∥\Lambda_{i}{∥}_{F}K_{gi}\epsilon_{xi}+2\lambda_{\text{max}}\left(P_{i}\right)∥B_{i}{∥}_{F}{∥\Lambda_{i}∥}_{F}\epsilon_{ui}+2\lambda_{\text{max}}\left(P_{i}\right)\left(∥B_{i}K_{1i}{∥}_{F}+{∥L_{i}∥}_{F}\right)\epsilon_{xi}\\ & +2{\tilde{w}}_{i}^{*}\lambda_{\text{max}}\left(P_{i}\right)∥B_{i}{∥}_{F}∥\Lambda_{i}{∥}_{F}\epsilon_{xi}]∥{\tilde{x}}_{i}\left(t\right)∥+\left[4l_{i}\lambda_{\text{min}}\left(R_{i}\right)\epsilon_{xi}+2{\tilde{w}}_{i}^{*}\lambda_{\text{max}}\left(P_{i}\right)∥B_{i}{∥}_{F}{∥\Lambda_{i}∥}_{F}\epsilon_{xi}\right]∥{\hat{e}}_{i}\left(t\right)∥\\ & +2{\tilde{w}}_{i}^{*}\lambda_{\text{max}}\left(P_{i}\right)∥B_{i}{∥}_{F}{∥\Lambda_{i}∥}_{F}\epsilon_{xi}\left(\left(L_{\beta i}+1\right)\left(\epsilon_{xi}+x_{ri}^{*}\right)+∥c_{i}\left(t\right)∥+\sum_{i∼j}L_{\phi ij}\left(\epsilon_{xj}+x_{rj}^{*}\right)\right)\\ & +2{\tilde{w}}_{i}^{*}\lambda_{\text{max}}\left(P_{i}\right)∥B_{i}{∥}_{F}{∥\Lambda_{i}∥}_{F}\epsilon_{xi}\sum_{i∼j}L_{\phi ij}\left(∥{\tilde{x}}_{j}\left(t\right)∥+∥{\hat{e}}_{j}\left(t\right)∥\right) \end{array}$$

It then follows that Equation (95) can be given by:

(96) $$\begin{array}{cc} & {\dot{V}}_{i}\left({\tilde{x}}_{i}\left(t\right),{\tilde{W}}_{i}\left(t\right),{\hat{e}}_{i}\left(t\right)\right)\\ & \leq \;-d_{1i}∥{\tilde{x}}_{i}{\left(t\right)∥}^{2}-d_{2i}∥{\hat{e}}_{i}{\left(t\right)∥}^{2}+d_{3i}∥{\tilde{x}}_{i}\left(t\right)∥+d_{4i}∥{\hat{e}}_{i}\left(t\right)∥+d_{5i}+F_{i}\sum_{i∼j}L_{\phi ij}∥{\tilde{x}}_{j}\left(t\right)∥\\ & +F_{i}\sum_{i∼j}L_{\phi ij}∥{\hat{e}}_{j}\left(t\right)∥ \end{array}$$

where $d_{1i}≜\lambda_{\text{min}}\left(R_{i}\right)$, $d_{2i}≜2l_{i}{∥L_{i}∥}_{F}^{-1}\lambda_{\text{max}}^{-1}\left(P_{i}\right)\lambda_{\text{min}}\left(R_{i}\right)\left[\lambda_{\text{min}}\left(R_{i}\right)-l_{i}\right]$, $d_{3i}≜2\lambda_{\text{max}}\left(P_{i}\right)∥B_{i}{∥}_{F}{∥\Lambda_{i}∥}_{F}K_{gi}\epsilon_{xi}$ $+2\lambda_{\text{max}}\left(P_{i}\right)∥B_{i}{∥}_{F}∥\Lambda_{i}{∥}_{F}\epsilon_{ui}+2\lambda_{\text{max}}\left(P_{i}\right)\left(∥B_{i}K_{1i}{∥}_{F}+{∥L_{i}∥}_{F}\right)\epsilon_{xi}+2{\tilde{w}}_{i}^{*}\lambda_{\text{max}}\left(P_{i}\right)∥B_{i}{∥}_{F}∥\Lambda_{i}{∥}_{F}\epsilon_{xi},\;d_{4i}≜4l_{i}\lambda_{\text{min}}\left(R_{i}\right)\cdot \epsilon_{xi}+2{\tilde{w}}_{i}^{*}\lambda_{\text{max}}\left(P_{i}\right)∥B_{i}{∥}_{F}{∥\Lambda_{i}∥}_{F}\epsilon_{xi}$, $d_{5i}≜2{\tilde{w}}_{i}^{*}\lambda_{\text{max}}\left(P_{i}\right)∥B_{i}{∥}_{F}{∥\Lambda_{i}∥}_{F}\epsilon_{xi}\left(\left(L_{\beta i}+1\right)\left(\epsilon_{xi}+x_{ri}^{*}\right)+∥c_{i}\left(t\right)∥+\sum_{i∼j}L_{\phi ij}\left(\epsilon_{xj}+x_{rj}^{*}\right)\right)$ and $f_{i}≜2{\tilde{w}}_{i}^{*}\lambda_{\text{max}}\left(P_{i}\right)∥B_{i}{∥}_{F}{∥\Lambda_{i}∥}_{F}\epsilon_{xi}$. To ensure that $d_{2i}$ is positive definite, we consider $l_{i}=\theta_{i}\lambda_{\text{min}}\left(R_{i}\right)$ and $\theta_{i}\in \left(0,1\right)$.

Introducing:

(97) $$\begin{array}{c} V\left(\cdot \right)=\sum_{i=1}^{N}V_{i}\left({\tilde{x}}_{i}\left(t\right),{\tilde{W}}_{i}\left(t\right){\hat{e}}_{i}\left(t\right)\right), \end{array}$$

for the uncertain system S results in:

(98) $$\begin{array}{cc} {\dot{V}}_{i}\left(\cdot \right) & \leq \sum_{i=1}^{N}[-d_{1i}∥{\tilde{x}}_{i}{\left(t\right)∥}^{2}-d_{2i}∥{\hat{e}}_{i}{\left(t\right)∥}^{2}+d_{3i}∥{\tilde{x}}_{i}\left(t\right)∥+d_{4i}∥{\hat{e}}_{i}\left(t\right)∥+d_{5i}+F_{i}\sum_{i∼j}L_{\phi ij}∥{\tilde{x}}_{j}\left(t\right)∥\\ & +F_{i}\sum_{i∼j}L_{\phi ij}∥{\hat{e}}_{j}\left(t\right)∥]\\ & =\sum_{i=1}^{N}\left[-d_{1i}∥{\tilde{x}}_{i}{\left(t\right)∥}^{2}-d_{2i}∥{\hat{e}}_{i}{\left(t\right)∥}^{2}+\underset{D_{3i}}{\underset{︸}{\left(d_{3i}+\sum_{i∼j}f_{j}L_{\phi ji}\right)}}∥{\tilde{x}}_{i}\left(t\right)∥+\underset{D_{4i}}{\underset{︸}{\left(d_{4i}+\sum_{i∼j}f_{j}L_{\phi ji}\right)}}∥{\hat{e}}_{i}\left(t\right)∥+d_{5i}\right] \end{array}$$

Letting ${\tilde{x}}_{a}\left(t\right)≜{\left[∥{\tilde{x}}_{1}\left(t\right)∥,\cdots ,∥{\tilde{x}}_{N}\left(t\right)∥\right]}^{T}$, ${\hat{e}}_{a}\left(t\right)≜{\left[∥{\hat{e}}_{1}\left(t\right)∥,\cdots ,∥{\hat{e}}_{N}\left(t\right)∥\right]}^{T}$, $D_{1}≜\text{diag}\left(\left[d_{11},\cdots ,d_{1N}\right]\right)$, $D_{2}≜\text{diag}\left(\left[d_{21},\cdots ,d_{2N}\right]\right)$, $D_{3}≜\text{diag}\left(\left[D_{31},\cdots ,D_{3N}\right]\right)$, $D_{4}≜\text{diag}\left(\left[D_{41},\cdots ,D_{4N}\right]\right)$, and $D_{5}≜\sum_{i=1}^{N}d_{5i}$, then Equation (98) can equivalently be written as:

(99) $$\begin{array}{cc} \dot{V}\left(\cdot \right) & \leq -{\tilde{x}}_{a}^{T}\left(t\right)D_{1}{\tilde{x}}_{a}\left(t\right)-{\hat{e}}_{a}^{T}\left(t\right)D_{2}{\hat{e}}_{a}\left(t\right)+D_{3}{\tilde{x}}_{a}\left(t\right)+D_{4}e_{a}\left(t\right)+D_{5}\\ & \leq -\lambda_{\text{min}}\left(D_{1}\right)∥{\tilde{x}}_{a}\left(t\right){∥}^{2}-\lambda_{\text{min}}\left(D_{2}\right)∥{\hat{e}}_{a}\left(t\right){∥}^{2}+\lambda_{\text{max}}\left(D_{3}\right)∥{\tilde{x}}_{a}\left(t\right)∥+\lambda_{\text{max}}\left(D_{4}\right)∥{\hat{e}}_{a}\left(t\right)∥+D_{5} \end{array}$$

Either $∥{\tilde{x}}_{a}\left(t\right)∥>\psi_{1}$ or $∥{\hat{e}}_{a}\left(t\right)∥>\psi_{2}$, renders $\dot{V}\left(\cdot \right)<0$, where $\psi_{1}≜\frac{\frac{\lambda_{\text{max}}\left(D_{3}\right)}{2\sqrt{\lambda_{\text{min}}\left(D_{1}\right)}}+\sqrt{\frac{\lambda_{\text{max}}^{2}\left(D_{3}\right)}{4\lambda_{\text{min}}\left(D_{1}\right)}+\frac{\lambda_{\text{max}}^{2}\left(D_{4}\right)}{4\lambda_{\text{min}}\left(D_{2}\right)}+D_{5}}}{\sqrt{\lambda_{\text{min}}\left(D_{1}\right)}}$ and $\psi_{2}≜\frac{\frac{\lambda_{\text{max}}\left(D_{4}\right)}{2\sqrt{\lambda_{\text{min}}\left(D_{2}\right)}}+\sqrt{\frac{\lambda_{\text{max}}^{2}\left(D_{3}\right)}{4\lambda_{\text{min}}\left(D_{1}\right)}+\frac{\lambda_{\text{max}}^{2}\left(D_{4}\right)}{4\lambda_{\text{min}}\left(D_{2}\right)}+D_{5}}}{\sqrt{\lambda_{\text{min}}\left(D_{2}\right)}}$, and hence, ${\tilde{x}}_{i}\left(t\right)$, ${\hat{e}}_{i}\left(t\right)$, and ${\tilde{W}}_{i}\left(t\right)$ are uniformly ultimate bounded for all i=1,2,...,N. ☐

**Remark 4.**  *To show that $e_{i}\left(t\right)$ is bounded for all i=1,2,⋯,N under the condition of Corollary 7, we can follow Corollary 4 to show the boundedness of $e_{i}\left(t\right)$ for all i=1,⋯,N using:*

(100) $$\begin{array}{c} ∥e_{i}\left(t\right)∥\leq ∥{\tilde{x}}_{i}\left(t\right)∥+∥{\hat{e}}_{i}\left(t\right)∥ \end{array}$$

Furthermore, in order to obtain the closed-loop system error ultimate bound value for Equation (100) and the no Zeno characterization proof, we can follow the same steps highlighted in Corollaries 5 and 6, respectively.

## 4. Illustrative Numerical Example

**Figure 2:**
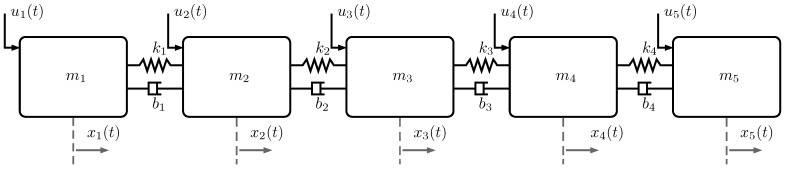
Connected mass-damper-spring system.

In this section, the efficacy of the proposed event-triggered decentralized adaptive control approach is demonstrated in an illustrative numerical example. For this purpose, we consider the uncertain dynamical system, which consists of five masses connected serially by springs and dampers as depicted in Figure 2. We use the following equations of motion for the i-th mass:

(101) $$\begin{array}{ccc} \left[\begin{array}{c} {\dot{x}}_{1}\left(t\right)\\ {\ddot{x}}_{1}\left(t\right) \end{array}\right] & = & \left[\begin{array}{cc} 0 & 1\\ \frac{-k_{1}}{m_{1}} & \frac{-b_{1}}{m_{1}} \end{array}\right]\left[\begin{array}{c} x_{1}\left(t\right)\\ {\dot{x}}_{1}\left(t\right) \end{array}\right]+\left[\begin{array}{c} 0\\ \frac{1}{m_{1}} \end{array}\right]\left[\Lambda_{1}u_{1}\left(t\right)+\Delta_{1}\left(x_{1}\left(t\right)\right)+\delta_{12}\left(x_{2}\left(t\right)\right)\right] \end{array}$$

(102) $$\begin{array}{c} \left[\begin{array}{c} {\dot{x}}_{i}\left(t\right)\\ {\ddot{x}}_{i}\left(t\right) \end{array}\right]=\left[\begin{array}{cc} 0 & 1\\ \frac{-(k_{i-1}+k_{i})}{m_{i}} & \frac{-(b_{i-1}+b_{i})}{m_{i}} \end{array}\right]\left[\begin{array}{c} x_{i}\left(t\right)\\ {\dot{x}}_{i}\left(t\right) \end{array}\right]+\left[\begin{array}{c} 0\\ \frac{1}{m_{i}} \end{array}\right]\left[\Lambda_{i}u_{i}\left(t\right)+\Delta_{i}\left(x_{i}\left(t\right)\right)+\delta_{ij}\left(x_{j}\left(t\right)\right)\right],\\ \;i=\left\{2,3,4\right\} \end{array}$$

(103) $$\begin{array}{ccc} \left[\begin{array}{c} {\dot{x}}_{5}\left(t\right)\\ {\ddot{x}}_{5}\left(t\right) \end{array}\right] & = & \left[\begin{array}{cc} 0 & 1\\ \frac{-k_{4}}{m_{5}} & \frac{-b_{4}}{m_{5}} \end{array}\right]\left[\begin{array}{c} x_{5}\left(t\right)\\ {\dot{x}}_{5}\left(t\right) \end{array}\right]+\left[\begin{array}{c} 0\\ \frac{1}{m_{5}} \end{array}\right]\left[\Lambda_{5}u_{i}\left(t\right)+\Delta_{5}\left(x_{5}\left(t\right)\right)+\delta_{54}\left(x_{4}\left(t\right)\right)\right] \end{array}$$

where $m_{i}=1$Kg, $k_{i}=1.5$ N·m${}^{-1}$, $b_{i}=0.4$ N·sec·m${}^{-1}$, $\Lambda_{i}=0.7$, $W_{oi}={\left[3\;,\;1\right]}^{T}$, and we set the basis function as $\beta_{i}\left(x_{i}\left(t\right)\right)=x_{i}\left(t\right)$. In addition, $\delta_{12}\left(x_{2}\left(t\right)\right)$, $\delta_{ij}\left(x_{j}\left(t\right)\right)$ and $\delta_{54}\left(x_{4}\left(t\right)\right)$, which represent the effect of the system interconnections, are given by:

(104) $$\begin{array}{ccc} \delta_{12}\left(x_{2}\left(t\right)\right) & = & [\begin{array}{cc} k_{1} & b_{1} \end{array}]\left[\begin{array}{c} x_{2}\left(t\right)\\ {\dot{x}}_{2}\left(t\right) \end{array}\right] \end{array}$$

(105) $$\begin{array}{c} \;\delta_{ij}\left(x_{j}\left(t\right)\right)=[\begin{array}{cc} k_{j=i-1} & b_{j=i-1} \end{array}]\left[\begin{array}{c} x_{j=i-1}\left(t\right)\\ {\dot{x}}_{j=i-1}\left(t\right) \end{array}\right]+[\begin{array}{cc} k_{j=i} & b_{j=i} \end{array}]\left[\begin{array}{c} x_{j=i+1}\left(t\right)\\ {\dot{x}}_{j=i+1}\left(t\right) \end{array}\right],\\ \;i=\left\{2,3,4\right\} \end{array}$$

(106) $$\begin{array}{ccc} \delta_{54}\left(x_{4}\left(t\right)\right)) & = & [\begin{array}{cc} k_{4} & b_{4} \end{array}]\left[\begin{array}{c} x_{4}\left(t\right)\\ {\dot{x}}_{4}\left(t\right) \end{array}\right] \end{array}$$

The control objective of each module is to enforce $x_{i}\left(t\right)$ to track a filtered square reference input $c_{i}\left(t\right)$ under the effect of uncertainties and disturbances with reduced communication effort by event-triggering architecture. For our example, we choose a second-order ideal reference model that has a natural frequency of 2 rad/s and a damping ratio of 0.707 for all $S_{i},i=1,\cdots ,5$. In addition, we use a state emulator gain $L_{i}=9I_{2}$ and set all initial conditions to zero for all $S_{i},i=1,\cdots ,5$.

For the event-triggered decentralized model reference adaptive control (which is equivalent to $L_{i}=0$), we set $Q_{i}=I_{2}$ in order to compute $P_{i}$ in Equation (10). The condition in Assumption 4 holds when $\alpha_{ij}\leq 0.26$ for $i=\left\{1,5\right\}$ and $\alpha_{ij}\leq 0.13$ for $i=\left\{2,3,4\right\}$. In this case, Assumption 2 is satisfied for the coupling terms given in Equations (104)–(106). For the purpose of event-triggered state emulator-based decentralized adaptive control, we set $R_{i}=3$ and $Q_{i}=I_{2\times 2}$ in order to compute $P_{i}$ in Equation (48). For $l_{i}=0.001$ and ${\tilde{Q}}_{0i}=250I_{2},$ the condition in Assumption 5 holds when $\alpha_{ij}\;\leq \;4.2$ for $i=\left\{1,5\right\}$ and $\alpha_{ij}\leq 2.1$ for $i=\left\{2,3,4\right\}$. In addition, Assumption 2 is satisfied for coupling terms given by Equations (104)–(106).

For the proposed event-triggered distributed adaptive control, we set $Q_{i}=I_{2}$ in order to compute $P_{i}$ in Equation (10). Note that there are no fundamental stability conditions for the case of distributed adaptive control. Lastly, for the event-triggering thresholds, we choose $\epsilon_{xi}=0.2$ and $\epsilon_{ui}=0.2$ for $i=\left\{1,3,5\right\}$ and $\epsilon_{xi}=0.07$ and $\epsilon_{ui}=0.07$ for $i=\left\{2,4\right\}$.

**Figure 3:**
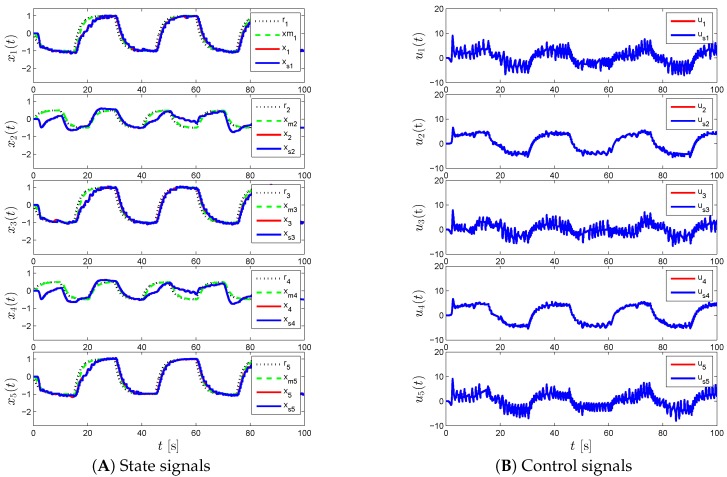
Command following performance for the proposed event-triggered decentralized adaptive control approach with *γ_i_* = 50 and *L_i_* = 0.

**Figure 4:**
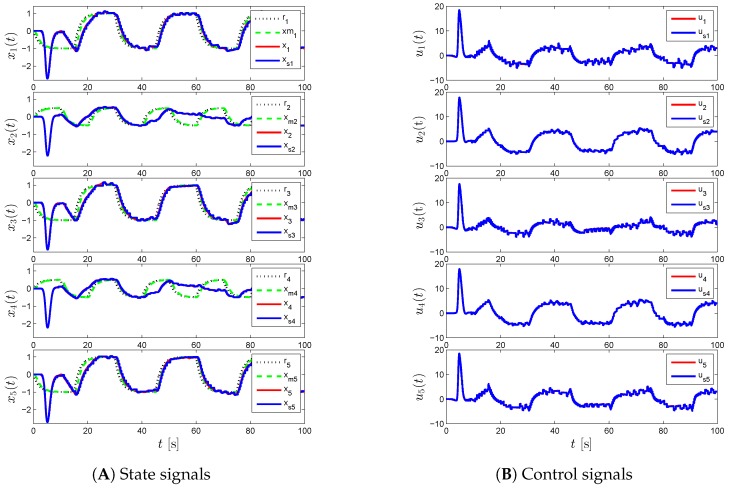
Command following performance for the proposed event-triggered decentralized adaptive control approach with *γ_i_* = 50 and *L_i_* = 9.

**Figure 5:**
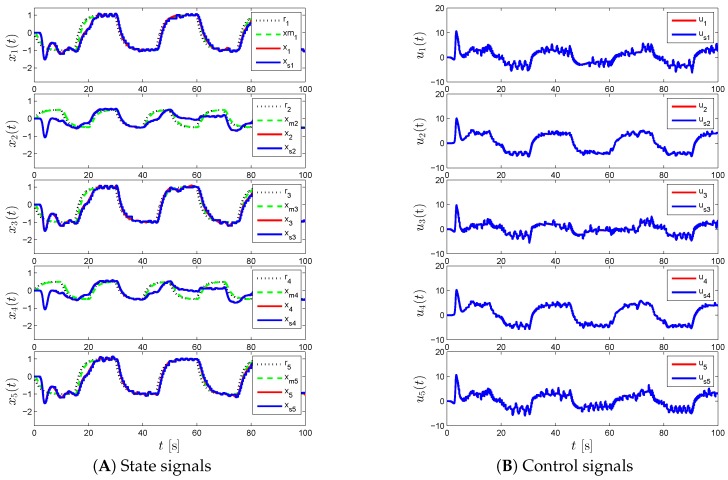
Command following performance for the proposed event-triggered decentralized adaptive control approach with *γ_i_* = 200 and *L_i_* = 9.

For the proposed event-triggered decentralized adaptive control design of Theorem 1 and Corollary 1, Figure 3, Figure 4 and Figure 5 represent the results for various $\gamma_{i}$ and $L_{i}$. In particular, we first set $\gamma_{i}=50$ and $L_{i}=0$ in Figure 3, which results in a control response with high-frequency oscillations. In order to suppress these undesired oscillations, we set $L_{i}=9I_{2}$ as seen in Figure 4. In this figure, even though such oscillations are reduced, the command tracking performance becomes worse as we increase $L_{i}$ compared to the response in Figure 3. In addition to increasing $L_{i}$, we also increase $\gamma_{i}$ in Figure 5, to improve command tracking performance without causing high-frequency oscillations. In general, if one picks $L_{i}$ to be greater than nine, then it may also be necessary to increase $\gamma_{i}$ further to obtain a similar closed-loop system performance. It should also be mentioned that choosing $L_{i}$ and $\gamma_{i}$ to produce both a control response without any significant high-frequency oscillations, and a small uniform ultimate bound can be cast as an optimization problem, as well.

**Figure 6:**
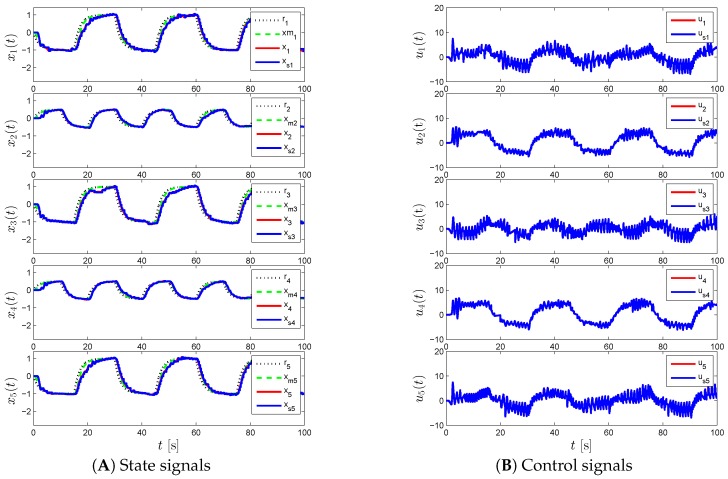
Command following performance for the proposed event-triggered distributed adaptive control approach with *γ_i_* = 50 and *L_i_* = 0.

**Figure 7:**
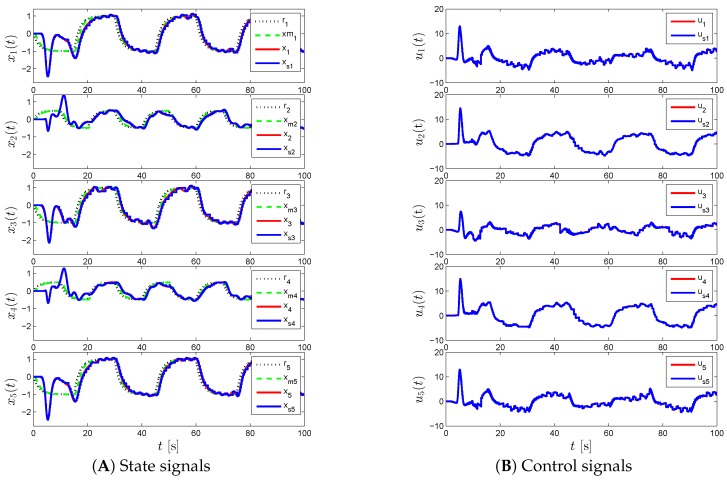
Command following performance for the proposed event-triggered distributed adaptive control approach with *γ_i_* = 50 and *L_i_* = 9.

**Figure 8:**
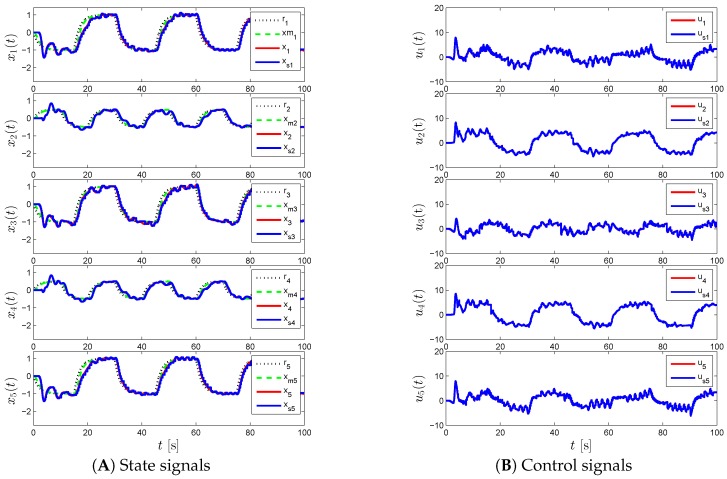
Command following performance for the proposed event-triggered distributed adaptive control approach with *γ_i_* = 200 and *L_i_* = 9.

Figure 6, Figure 7 and Figure 8 represent the results of the proposed event-triggered distributed adaptive control of Theorem 2 and Corollary 7 for the same $\gamma_{i}$ and $L_{i}$ values. Specifically, we see high frequency content in the control signal in Figure 6 when $\gamma_{i}=50$ and $L_{i}=0$, which is mitigated by increasing the state emulator gain to $L_{i}=9I_{2}$, as seen in Figure 7. In order to enhance the command tracking, which is degraded by increasing the state emulator gain, we increase $\gamma_{i}$ as seen in Figure 8.

**Figure 9:**
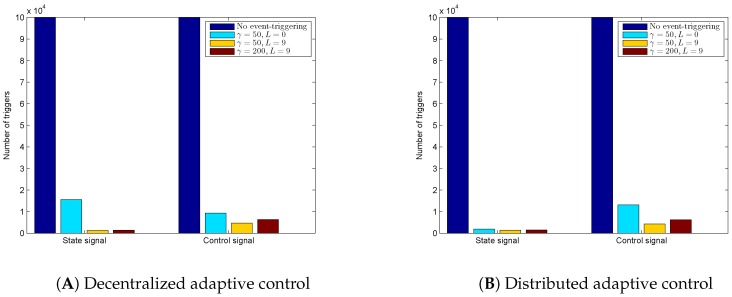
Number of triggers with respect to the controller design parameters.

From these results, we observe from the decentralized adaptive control case that the state emulator-based approach not only gives stringent performance without causing high frequencies in the controller response, but also tolerates the interconnection uncertainties of the modules. In addition, the performance of the distributed adaptive controller is better than the decentralized adaptive controller with the corresponding design parameter setting. The total number of the state and control event triggers of the whole system for the cases in Figure 3, Figure 4, Figure 5, Figure 6, Figure 7 and Figure 8 is given in Figure 9A,B, respectively. Figure 9 shows the drastic decrement of the triggering number using the event-triggering approach and also the further triggering number decrement due to utilizing the state emulator-based approach.

## 5. Conclusions

The design and analysis of event-triggered decentralized and distributed adaptive control architectures for uncertain networked large-scale modular systems were presented. For the decentralized case, it was shown in Section 2 that the proposed event-triggered adaptive control architecture guarantees system stability and performance with no Zeno behavior under some structural conditions stated in Assumptions 4 and 5 that depend on the parameters of the large-scale modular systems and the proposed architecture. For the distributed case, it was shown in Section 3 that the proposed event-triggered adaptive control architecture guarantees the same system stability and performance with no Zeno behavior without such structural conditions under the assumption that physically-interconnected modules can locally communicate with each other for exchanging their state information. In addition to the presented theoretical findings, the efficacy of the proposed event-triggered decentralized and distributed adaptive control approaches is demonstrated on an illustrative numerical example in Section 4, where significant reduction on the overall communication cost was obtained for large-large modular systems in the presence of system uncertainties resulting from modeling and degraded modes of operation of the modules and their interconnections between each other. For the future work, sampling, data transmission and computation delays will be considered along with the proposed results of this paper, since they also play an important role in the performance of networked control systems. Furthermore, we will also consider the cases when a set of diagonal elements of the control effectiveness matrix is zero and generalize the results of this paper to cover these so-called loss of control cases.
